# Clinician Job Satisfaction After Peer Comparison Feedback: A Secondary Analysis of a Randomized Clinical Trial

**DOI:** 10.1001/jamanetworkopen.2023.17379

**Published:** 2023-06-08

**Authors:** Jason N. Doctor, Noah J. Goldstein, Craig R. Fox, Jeffrey A. Linder, Stephen D. Persell, Emily P. Stewart, Tara K. Knight, Daniella Meeker

**Affiliations:** 1 Leonard D. Schaeffer Center for Health Policy and Economics, University of Southern California, Los Angeles; 2 UCLA Anderson School of Management, UCLA Geffen School of Medicine, Los Angeles, California; 3 Division of General Internal Medicine, Northwestern University Feinberg School of Medicine, Chicago, Illinois; 4 Department of Biomedical Informatics & Data Science, Yale University School of Medicine, New Haven, Connecticut

## Abstract

**Importance:**

Interventions that improve clinician performance through feedback should not contribute to job dissatisfaction or staff turnover. Measurement of job satisfaction may help identify interventions that lead to this undesirable consequence.

**Objective:**

To evaluate whether mean job satisfaction was less than the margin of clinical significance among clinicians who received social norm feedback (peer comparison) compared with clinicians who did not.

**Design, Setting, and Participants:**

This secondary, preregistered, noninferiority analysis of a cluster randomized trial compared 3 interventions to reduce inappropriate antibiotic prescribing in a 2 × 2 × 2 factorial design from November 1, 2011, to April 1, 2014. A total of 248 clinicians were enrolled from 47 clinics. The sample size for this analysis was determined by the number of nonmissing job satisfaction scores from the original enrolled sample, which was 201 clinicians from 43 clinics. Data analysis was performed from October 12 to April 13, 2022.

**Interventions:**

Feedback comparing individual clinician performance to top-performing peers, delivered in monthly emails (peer comparison).

**Main Outcomes and Measures:**

The primary outcome was a response to the following statement: “Overall, I am satisfied with my current job.” Responses ranged from 1 (strongly disagree) to 5 (strongly agree).

**Results:**

A total of 201 clinicians (response rate, 81%) from 43 of the 47 clinics (91%) provided a survey response about job satisfaction. Clinicians were primarily female (n = 129 [64%]) and board certified in internal medicine (n = 126 [63%]), with a mean (SD) age of 48 (10) years. The clinic-clustered difference in mean job satisfaction was greater than −0.32 (β = 0.11; 95% CI, −0.19 to 0.42; *P* = .46). Therefore, the preregistered null hypothesis that peer comparison is inferior by resulting in at least a 1-point decrease in job satisfaction by 1 in 3 clinicians was rejected. The secondary null hypothesis that job satisfaction was similar among clinicians randomized to social norm feedback was not able to be rejected. The effect size did not change when controlling for other trial interventions (*t* = 0.08; *P* = .94), and no interaction effects were observed.

**Conclusions and Relevance:**

In this secondary analysis of a randomized clinical trial, peer comparison did not lead to lower job satisfaction. Features that may have protected against dissatisfaction include clinicians’ agency over the performance measure, privacy of individual performance, and allowing all clinicians to achieve top performance.

**Trial Registration:**

ClinicalTrials.gov Identifiers: NCT05575115 and NCT01454947

## Key Points

**Question:**

Does receipt of peer comparison (also known as social norm or relative performance) feedback on antibiotic prescribing have a significant negative impact on job satisfaction among primary care clinicians in Boston, Massachusetts, and Los Angeles, California?

**Findings:**

In this secondary analysis of a randomized clinical trial conducted from November 1, 2011, to April 1, 2014, the intervention, which delivered a comparison of individual clinician performance with that of top-performing peers in monthly emails, did not reduce mean job satisfaction beyond the noninferiority margin of clinical significance.

**Meaning:**

These findings suggest that behavioral interventions aimed at improving performance can be designed in a way that may protect clinician job satisfaction.

## Teaser

This secondary analysis of a randomized clinical trial examines whether a national randomized trial using peer comparison feedback to change antibiotic-prescribing behavior has a significant negative impact on physician job satisfaction.

## Introduction

Many performance improvement interventions that exert social influence have shown early signs of success,^1,2,3^ but their effect on clinician well-being and the risk of job turnover is not well understood. Clinician well-being is a national concern. Physicians have less satisfaction and higher rates of burnout than the general workforce.^4^ Lower job satisfaction in medicine predicts intent to reduce clinical hours, intent to leave, and leaving one’s current practice.^5,6^ Clinician shortages on the horizon may also lead to a greater burden on physicians and even lower well-being.^7^ Interventions that improve the quality of care need to do so without harming job satisfaction. Peer comparison feedback could affect job satisfaction if the intervention was perceived as a form of undue pressure to improve performance. This study aimed to evaluate whether a national randomized clinical trial using social norm feedback to change antibiotic prescribing behavior (feedback based on a comparison with top-performing peers) had a significant negative impact on job satisfaction.

## Methods

This report follows the Consolidated Standards of Reporting Trials (CONSORT) reporting guideline for randomized clinical trials.^8^ The study was also reviewed and approved by the University of Southern California Institutional Review Board. Written informed consent was obtained from all participants.

### Study Design and Participants

The Use of Behavioral Economics and Social Psychology to Improve Treatment of Acute Respiratory Infections Trial was a multisite, cluster randomized trial with practice as the unit of randomization.^9,10^ The primary aim was to test whether 3 interventions based on behavioral economic principles reduced the rate of inappropriate antibiotic prescribing for acute respiratory tract infections in a 2 × 2 × 2 factorial design. Because of the factorial structure of the design, half the participants were randomized to peer comparison and the other half were not (see Supplement 1 for the trial protocol).

A total of 248 participants (practicing attending clinicians or advanced practice nurses) were enrolled from 47 participating outpatient clinic sites in Boston, Massachusetts, and Los Angeles, California. Prescribing data from electronic medical records for participating practices were transferred to the data coordinating center on a weekly basis. Data on race/ethnicity were not collected. Surveys were administered using Qualtrics, version XM (Qualtrics); up to 3 survey reminders were sent by email.

### Intervention

Peer comparison feedback was delivered in monthly email messages from practice leadership during the 18-month intervention period. We assessed clinicians’ inappropriate prescribing rates within each geographic region using electronic health record data. Clinicians ranked in the lowest decile of inappropriate prescribing rates (the top-performing decile) were told via email that they were a “top performer.” The remaining clinicians were told that they were “not a top performer.” Both types of emails included the number and proportion of antibiotic prescriptions the clinician wrote for antibiotic-inappropriate acute respiratory tract infections. Those labeled “not top performers” were also shown the proportion written by top performers. Ties in rankings were allowed so any number of clinicians could be labeled “top performers.”

### Measurement

Exit surveys, which included a question about job satisfaction, were launched 1 to 12 weeks (varying by institution) following the 18-month intervention. Thereafter, clinicians received up to 3 reminders to complete their survey by the end of 8 weeks; completion times were the same between clinicians who did and did not receive social norm feedback because randomization was stratified by institution. Responses to the following statement were measured on a 5-point Likert scale: “Overall, I am satisfied with my current job.” Responses ranged from 1 (strongly disagree) to 5 (strongly agree).

### Hypothesis Testing

We preregistered a noninferiority hypothesis test (NCT05575115). We defined a clinically significant detrimental effect on job satisfaction as 33% of individuals reducing job satisfaction ratings by 1 point on a 5-point Likert scale for nonmissing responses greater than 1. This shift is equivalent to a mean difference of 0.32.

Our null hypothesis is that the peer comparison intervention^9^ had a clinically and statistically significant detrimental effect on clinician job satisfaction beyond the margin of clinical significance^11^: μ_Peer Comparison_ − μ_Control_ ≤ −0.32. Our alternative hypothesis is that the peer comparison intervention had no clinically significant negative impact on clinician job satisfaction: μ_Peer Comparison_ − μ_Control_ > −0.32. We conducted a traditional hypothesis test, μ_Peer Comparison_ − μ_Control_ = 0.00, as a secondary analysis.

### Sample Size

This study is a prespecified secondary analysis using data from a randomized clinical trial conducted from November 1, 2011, to April 1, 2014. A total of 248 clinicians were enrolled from 47 clinics. The sample size for this analysis was determined by the number of nonmissing job satisfaction scores from the original enrolled sample, which was 201 clinicians from 43 clinics. Assuming no interaction between interventions, an 80% response rate, at least 90% clinic participation, and an intraclinic correlation of 0.07, we have 80% power to reject the hypothesis that the intervention reduces job satisfaction on average by at least one-third of a Likert point.

### Statistical Analysis

Data analysis was performed from October 12 to April 13, 2022. Analyses were completed using R, version 3.6.0 (R Project for Statistical Computing), Stata, version 16 (StataCorp LLC), and SAS, version 9.4 (SAS Institute Inc). We used the regress command in Stata to test the difference in mean job satisfaction. We also preregistered a standard null hypothesis of no difference in job satisfaction as a secondary analysis. We explored the effect of peer comparison controlling for accountable justifications and suggested alternatives and concurrent interventions using separate 2- and 3-way interaction models. We conducted a 2-tailed *t* test using Stata’s lincom command to test whether the effect changed controlling for suggested alternatives and accountable justifications. All models were clustered by clinic with robust standard errors. A 2-sided *P* < .05 was considered statistically significant.

## Results

**Figure 1.**
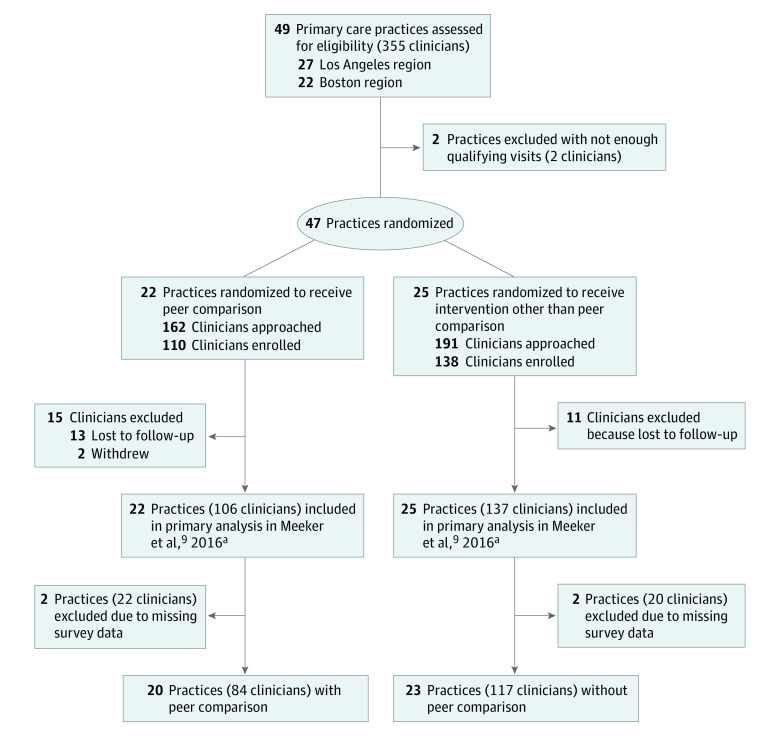
Participant Flow Diagram ^a^Most clinicians who were lost to follow-up were included in the primary intention-to-treat analysis. The number of clinicians in the primary analysis plus the number excluded will not add to the total number of clinicians enrolled.

The participant response rate was 81% (201 of 248), and the clinic participation rate was 91% (43 of 47) (Figure 1). The mean (SD) clinician age was 48 (10) years; 129 participants (64%) were female and 72 (36%) were male; and 126 (63%) were board certified in internal medicine. Participants’ mean (SD) time in practice was 20 (9) years (Table). Nonrespondents had a lower mean full-time equivalent (3.79; *P* < .001) but otherwise did not differ by patient and clinic characteristics.

**Table.** Practice and Clinician Characteristics

Characteristics	Overall	Peer comparison	No peer comparison
Total practices, No. (%)	43 (100)	20 (47)	23 (53)
Total clinicians, No. (%)	201 (100)	84 (42)	117 (58)
Age, mean (SD), y	48 (10)	49 (10)	48 (10)
Sex, No. (%)			
Female	129 (64)	50 (60)	79 (68)
Male	72 (36)	34 (40)	38 (32)
Time since first licensure, mean (SD), y	20 (9)	20 (9)	19 (10)
Time since first licensure, median (IQR), y	19 (12-27)	19 (13-27)	18 (12-26)
Clinician type, No. (%)			
Internal medicine	126 (63)	49 (58)	77 (66)
Family medicine	26 (13)	15 (18)	11 (9)
Other PCP	13 (6)	4 (5)	9 (8)
Physician assistant or nurse practitioner	16 (8)	9 (11)	7 (6)
Per-clinician FTE during intervention			
Mean (SD)	0.69 (0.32)	0.67 (0.30)	0.7 (0.30)
Median (IQR)	0.71 (0.49-1.00)	0.67 (0.45-0.93)	0.71 (0.49-1.00)
Total clinicians by region, No. (%)			
Massachusetts	143 (71)	56 (67)	87 (74)
Southern California	58 (29)	28 (33)	30 (26)

Abbreviations: FTE, full-time equivalent; PCP, primary care practice.

**Figure 2.**
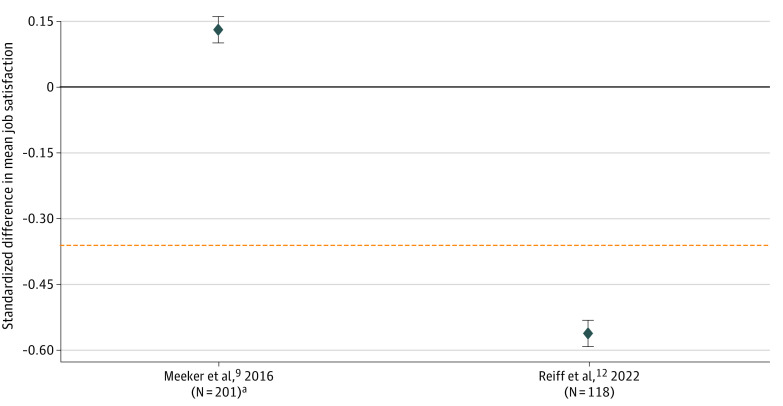
Standardized Mean Job Satisfaction The orange dashed line represents the null hypothesis. Whiskers represent 95% CIs. ^a^Forty-seven enrolled clinicians from 4 clinics were excluded from the analysis because of missing survey responses.

Mean (SD) job satisfaction was 3.73 (0.88). Prescribers randomized to peer comparison had 0.11 (95% CI, −0.19 to 0.42; *P* = .46) greater mean job satisfaction compared with control prescribers. We reject the hypothesis that peer comparison is detrimental to job satisfaction because we have 95% confidence that the point estimate 0.11 does not exceed the prespecified −0.32 threshold. We do not reject our secondary null hypothesis that peer comparison provides equal job satisfaction relative to other interventions. Reiff et al^12^ found that peer comparison feedback decreased unclustered, standardized mean job satisfaction by −0.56, beyond our margin of clinical significance. Figure 2 compares our estimate with those of Reiff et al.^12^

The effect of peer comparison on job satisfaction did not change after adjusting for accountable justifications and suggested alternatives (*t* = 0.08; *P* = .94) (eTable 1 in Supplement 2). Job satisfaction was also not affected when we included 2- and 3-way interaction terms among treatments (eTables 2 and 3 in Supplement 2).

## Discussion

Performance feedback using peer comparison is a widely used approach in health and public policy to change behavior. We rejected the hypothesis that there were negative effects on job satisfaction after a peer comparison intervention. We note that a previous study^12^ found that using peer comparison in primary care was ineffective and reduced job satisfaction. In that study,^12^ a bundle of metrics was used for rankings, some with limited clinician control over the outcomes; there was public disclosure of performance; and a restricted number of individuals could achieve the highest status among clinicians. In contrast, the trial^9^ used in the current analysis gave clinicians full agency over the outcome, kept performance private, did not restrict the number of top performers, and was successful in improving clinician behavior without lowering job satisfaction.

### Limitations

This study has some limitations. The primary limitation is that it is a secondary analysis of a randomized clinical trial. Additionally, participant enrollment and nonresponse to the survey may have introduced selection effects, although study enrollment (70%) and the survey response rate (81%) were relatively high. Survey response latency may have affected perceptions of the intervention. Another limitation is that the survey question was a single, one-time measure that may not accurately reflect job satisfaction.

## Conclusions

The conflicting findings between the 2 studies suggest that the details of how peer comparison is implemented may be crucial for its success and impact on clinician well-being.^13^ It is possible that clinician performance and job satisfaction benefit from having complete agency over the peer comparison measure. In addition, those implementing the intervention may need to keep individual performance information private and allow all clinicians an opportunity to achieve the highest level of performance. Some or all of these features may protect against clinician dissatisfaction reported elsewhere.^12^ Future work should focus on qualitative assessments to better understand which elements of social norm feedback are detrimental to or supportive of a clinician’s job satisfaction.

## Notes

Supplement 1.
Trial Protocol

Supplement 2. **eTable 1.** Linear Model Estimating Effect of Each Intervention on Job Satisfaction **eTable 2. **Two-Way Interaction Linear Model Estimating Effect of Peer Comparison on Job Satisfaction When Combined With the Accountable Justification or Suggested Alternatives Intervention **eTable 3. **Three-Way Interaction Model Estimating Effect of Peer Comparison on Job Satisfaction When Combined With the Accountable Justification and Suggested Alternatives Intervention

Supplement 3.
Data Sharing Statement
