# Supplementary material for: Clinician Job Satisfaction After Peer Comparison Feedback: A Secondary Analysis of a Randomized Clinical Trial
Source: JAMA Netw Open. 2023 Jun 8;6(6):e2317379. doi: 10.1001/jamanetworkopen.2023.17379 (PMC10251208; doi:10.1001/jamanetworkopen.2023.17379)
Supplement: Supplement 1. — Trial Protocol [file jamanetwopen-e2317379-s001.pdf]

STUDY PROTOCOL

Use of Behavioral Economics to Improve Treatment of Acute Respiratory Infections (BEARI): A cluster randomized controlled trial

Principal Investigator:

Jason Doctor, Ph.D., Associate Professor, University of Southern California

Supported by:

The National Institute on Aging

1RC4AG039115-01

Version 2
Date: March 14, 2013

Changes since Version 1 (version date: August 1, 2011): Intervention names were modified as follows: Alternative Prescriptions became Suggested Alternatives, Justification Alert became Accountable Justification, and Social Norms became Peer Comparison.

TABLE OF CONTENTS

STUDY TEAM ROSTER **6**

PARTICIPATING STUDY SITES **7**

PRÉCIS **8**

1. Study objectives **10**

1.1 Primary Objective 10

1.2 Secondary Objectives 10

2. BACKGROUND AND RATIONALE **10**

2.1 Background on Condition, Disease, or Other Primary Study Focus 10

2.2 Study Rationale 10

3. STUDY DESIGN **12**

4. SELECTION AND ENROLLMENT OF PARTICIPANTS **13**

4.1 Inclusion Criteria 13

4.2 Exclusion Criteria 13

4.3 Study Enrollment Procedures 13

5. STUDY INTERVENTIONS **14**

5.1 Interventions, Administration, and Duration 14

5.2 Handling of Study Interventions 14

5.3 Adherence Assessment 14

6. STUDY PROCEDURES **15**

6.1 Schedule of Evaluations 15

6.2 Description of Evaluations 17
6.2.1 Screening Evaluation 17
6.2.2 Enrollment, Baseline, and/or Randomization 17

7. SAFETY ASSESSMENTS 17

7.1 Specification of Safety Parameters 18

7.2 Methods and Timing for Assessing, Recording, and Analyzing Safety Parameters 18

7.3 Adverse Events 18

7.4 Reporting Procedures 18

7.5 Safety Monitoring 18

8. INTERVENTION DISCONTINUATION 19

9. STATISTICAL CONSIDERATIONS 19

9.1 General Design Issues 19

9.2 Sample Size and Randomization 20
9.2.1 Treatment Assignment Procedures 21

9.3 Interim analyses and Stopping Rules 22

9.4 Outcomes 22
9.4.1 Primary outcome 22
9.4.2 Secondary outcomes 22

9.5 Data Analyses 22

10. DATA COLLECTION AND QUALITY ASSURANCE 23

10.1 Data Collection Forms 23

10.2 Data Management 23

10.3 Quality Assurance 23
10.3.1 Training 23

10.3.2 Quality Control Committee 23
10.3.3 Metrics 24
10.3.4 Protocol Deviations 24
10.3.5 Monitoring 24

11. PARTICIPANT RIGHTS AND CONFIDENTIALITY 24

11.1 Institutional Review Board (IRB) Review 24

11.2 Informed Consent Forms 24

11.3 Participant Confidentiality 24

11.4 Study Discontinuation 25

12. COMMITTEES 25

13. PUBLICATION OF RESEARCH FINDINGS 25

14. REFERENCES 25

15. SUPPLEMENTS/APPENDICES 27

Table 1. Acute Respiratory Infection Diagnoses Related to Interventions and Outcomes Assessments 27

Appendix A: Survey and sample educational module at start of study 28

Appendix B: Post-study survey 45

Appendix C : Details of development and customization that was required at each site 54

Appendix D: Example of Accountable Justification decision support 56

Appendix E: Diagnosis code sets used in outcome assessments and clinical decision support 57

Appendix F: Example of Suggested Alternatives order set 58

Appendix G: Sample Peer Comparison emails to providers **60**

Appendix H: Oral antibiotics included in outcome measurements **61**

STUDY TEAM ROSTER

Jason N. Doctor, Ph.D. (Principal Investigator), Leonard D. Schaeffer Center for Health Policy and Economics, University of Southern California, 3335 S. Figueroa Street, Unit A, Los Angeles, CA 90089-7273, Office: 213.821.7943, Fax: 213.740.3460, jdoctor@usc.edu

Daniella Meeker, Ph.D (Co-Investigator), RAND, 1776 Main St, Santa Monica, CA 90401, dmeeker@rand.org

Stephen D. Persell, MD, MPH, .(Co-Investigator), Division of General Internal Medicine and Geriatrics, Institute for Healthcare Studies, Feinberg School of Medicine, Northwestern University, 750 N. Lake Shore Drive, 10th Floor, Chicago, IL 60611, spersell@nmff.org

Mark Friedberg, MD, MPP (Co-Investigator), RAND, 20 Park Plaza, Suite 920, Boston, MA 02116, mfriedbe@rand.org

Jeffrey A. Linder, MD, MPH, FACP (Co-Investigator), Division of General Medicine and Primary Care, Brigham and Women's Hospital, 1620 Tremont Street, BC-3-2X, Boston, MA 02120, jlinder@partners.org

Craig R. Fox, PhD (Co-Investigator), UCLA Anderson School of Management, 110 Westwood Plaza D-511, Los Angeles, CA 90095, craig.fox@anderson.ucla.edu

Noah J. Goldstein, Ph.D. (Co-Investigator), UCLA Anderson School of Management, 110 Westwood Plaza, A-412, Los Angeles, CA 90095, noah.goldstein@anderson.ucla.edu

Rick Chesler (Study Contact), Leonard D. Schaeffer Center for Health Policy and Economics
University of Southern California, 3335 S. Figueroa Street, Unit A, Los Angeles, CA 90089-7273, Office: 213.821.xxxx., Fax: 213.740.3460, rchesler@usc.edu

Tara K. Knight, Ph.D. (Project Manager) Leonard D. Schaeffer Center for Health Policy and Economics, University of Southern California, 3335 S. Figueroa Street, Unit A, Los Angeles, CA 90089-7273, Office: 213.821.7943, Fax: 213.740.3460, knight@usc.edu

PARTICIPATING STUDY SITES

Northwestern University. Stephen D. Persell, MD, MPH, Division of General Internal Medicine and Geriatrics, Institute for Healthcare Studies, Feinberg School of Medicine, Northwestern University, 750 N. Lake Shore Drive, 10th Floor, Chicago, IL 60611, spersell@nmff.org

Brigham and Women's Hospital. Jeffrey A. Linder, MD, MPH, FACP, Division of General Medicine and Primary Care, Brigham and Women's Hospital, 1620 Tremont Street, BC-3-2X, Boston, MA 02120, jlinder@partners.org

Massachusetts General Hospital. Carolyn Birks, MD, 15 Parkman St #555, Boston, MA 02114, 617-724-6610; cbirks@partners.org

AltaMed Health Services. Michael E. Hochman, MD, MPH, Medical Director for Innovation, 240 Camfield Ave, Los Angeles, CA 90040, 323 720 5676, mhochman@la.altamed.org

The Children's Clinic. Maria Chandler, MD, MBA, Chief Medical Officer, 2790 Atlantic Avenue, Long Beach, CA, 90806, 562-933-0446; mchandler@thechildrensclinic.org

PRÉCIS

Study Title

Use of Behavioral Economics and Social Psychology to Improve Treatment of Acute Respiratory Infections (BEARI): A cluster randomized controlled trial [1RC4AG039115-01]

Objectives

The main intent of this study is to determine whether interventions that leverage information technology and apply behavioral economic concepts reduce the rate of antibiotic prescribing for ARIs. Our primary hypothesis is that practices randomized to receive behavioral economic interventions will have lower antibiotic prescribing rates for non-antibiotic appropriate ARIs compared to control practices. We further hypothesize that for the treatment conditions, individual prescribers' rates of antibiotic prescribing for encounters with a non-antibiotic appropriate ARI diagnoses will decrease relative to their own historical control rates.

Design and Outcomes

We will conduct a multi-site cluster randomized trial of 3 behavioral interventions targeting unnecessary antibiotic prescribing during ambulatory visits for acute respiratory infections (ARIs), with practice as the unit of randomization.

The main outcome will be the rate of antibiotic prescribing for ambulatory visits for acute respiratory infections in which antibiotics are inappropriate (i.e., prescribing an antibiotic is inconsistent with published guidelines).

In the main analysis, we will compare a 6-month baseline period to an 18-month intervention period. In subsequent secondary analysis, we will track antibiotic prescribing for another 12 months after the interventions end.

Interventions and Duration

The intervention period will be 18-months in length for all participants, with a one year follow-up period to measure persistence of effects after interventions end. The following interventions will be compared: *Accountable Justification (AJ)* (also known as "Justification Alerts," or JA) is an EHR-based intervention which will prompt the clinician to justify, in a free text response, the decision to prescribe an antibiotic for each ARI. The prompt is designed to inform the clinician that the justification will be seen by others in the patient's medical record as an "Antibiotic Justification" note, and that if no justification is entered, the phrase "no justification given" will appear in the note. *Suggested Alternatives (SA)* is an EHR-based intervention most closely resembling

traditional clinical decision support alerts and order sets. ARI diagnoses will trigger a pop-up screen that states, "Antibiotics are not generally indicated for [this diagnosis]. Please consider the following prescriptions, treatments, and materials to help your patient." The screen will then suggest a list of alternatives see Appendix D: Example of Suggested Alternatives order set), each with a streamlined order entry option, such as for over-the-counter and prescription medications (e.g., decongestants) and letter templates excusing patients from work. *Peer Comparison (PC)* is an email-based intervention. Clinicians will be ranked from highest to lowest inappropriate prescribing rate within each region using EHR data. Clinicians with the lowest inappropriate prescribing rates (the top-performing 10th percentile) will be informed that they are a "Top Performer" in a congratulatory email. The remaining clinicians will be told that they are "Not a Top Performer" by email. Emails will include the number and proportion of inappropriate antibiotic prescriptions written for a month for non-antibiotic-appropriate ARI cases and the proportion written by Top Performers.

Sample Size and Population

We will recruit physicians and advance practice nurses from 49 primary care clinics affiliated with three healthcare organizations who see acute respiratory infection patients. Approximately 300 eligible providers seeing acute respiratory patients will be recruited for this study.

We will randomize practices (blocking on geographical region) to 0, 1, 2, or 3 interventions in a 2x2x2 factorial design to avoid contamination between individual clinicians within the same practice.

1. STUDY OBJECTIVES

1.1 Primary Objective

Our primary hypothesis is that practices randomized to receive behavioral economic interventions will have lower antibiotic prescribing rates for non-antibiotic appropriate ARIs compared to control practices.

1.2 Secondary Objectives

Secondary outcomes will examine antibiotic prescribing more broadly (including ARI diagnoses for which antibiotics might be appropriate), and extend the analysis for another 12 months of follow-up to investigate persistence of effects.

2. BACKGROUND AND RATIONALE

2.1 Background on Condition, Disease, or Other Primary Study Focus

Acute respiratory infections (ARIs) constitute about 10% of all ambulatory care visits in the United States and account for 44% all antibiotic prescriptions provided in ambulatory care.[1] Despite the fact that the vast majority of ARIs in adults are caused by viruses, antibiotic use for ARIs remains common.[1,2] Although the Centers for Disease Control and Prevention and others have placed increased emphasis on reducing inappropriate antibiotic use, prescribing rates declined only modestly between 1995 and 2006, and the use of broader-spectrum antibiotics increased.[1]

Clinicians who prescribe antibiotics for non-bacterial infections expose patients to unnecessary risks of adverse drug events, and increased costs.[3] Furthermore, antibiotic overuse increases the spread of antibiotic-resistant bacteria which have become a major public health problem.[2,4,5] Educational interventions may have limited impact on prescribing rates if lack of guideline awareness is not the primary reason for inappropriate antibiotic prescribing.

Recognizing the limitations of educational and informational interventions, we have developed novel interventions, drawing on insights from behavioral economics and social psychology, designed to appeal to provider self-image and social motivation and thereby produce larger and more enduring effects. These interventions take into account a growing body of research indicating that individuals act within broad social contexts and behave in ways that are not always rational but may be predictable.

2.2 Study Rationale

Rationale for Accountable Justifications. In the Accountable Justifications intervention, clinicians will be prompted to record an explicit justification for why they are prescribing an antibiotic to a patient with an ARI that appears in the patient's EHR. Accountable justifications incorporate several behavioral principles. First, they signal an injunctive norm (a norm, often provided by an authoritative source, that strongly indicates how people should behave) indicating that prescribing an antibiotic is not recommended. This may make the provider more likely to believe both that not prescribing an antibiotic is the best medical decision and that prescribing when it is not indicated violates professional standards. Second, they incorporate social accountability. Provider justifications become an explicit, separate part of the medical record, so a provider's decision to prescribe is subject to the review and judgment of the provider's peers.[6] Third, the justification alert implicitly designates guideline-concordant prescribing as the default action. Defaults are options that are exercised if the decision maker takes no special action to opt in or out of a given choice.[7-9] Prior to our intervention, choosing to deviate from guidelines did not carry a special requirement to document a clinical rationale in the EHR. Accountable justifications, therefore, reset the default action. Guideline-concordant treatment choices (i.e., not prescribing an antibiotic for an ARI) will continue to require no special justification, but a provider must now "opt-in" to prescribing an antibiotic by providing a justification for which they are accountable.

Rationale for Suggested Alternatives. When clinicians assigned to the Suggested Alternatives intervention see a patient with an ARI, they will receive a list of non-antibiotic treatment choices prior to the time when they would complete an antibiotic prescription. Suggested Alternatives may be effective because one central reason why physicians prescribe antibiotics for ARIs when they are not indicated is perceived pressure from patients requesting a prescription. Patients may be unsatisfied if they do not receive an antibiotic prescription, or at least a prescription for medication of some kind.[10] By making prescription and over-the-counter medications that are alternatives to antibiotics more salient to providers, we facilitate a means by which they can satisfy patient demand for treatment from their provider while at the same time reducing their tendency to prescribe unnecessary antibiotics.[9]

Rationale for Peer Comparisons. Social norms are standards that are understood by members of a group and that guide relevant social behavior due to a desire to conform with actual behavior (the descriptive norm) or sanctioned behavior (the injunctive norm).[11] Numerous studies have shown that people tend to conform to the behavior of others, especially those who are perceived to be similar to one's self.[12] Such effects have been found in studies of behaviors as diverse as voting, littering,[11] and towel recycling in hotels.[13] Social norms may convey information concerning appropriate behavior or social consequences of failing to conform. Behavioral studies find that these effects persist even when behavior is unobservable (e.g., littering when nobody is around) and when the social information is not particularly informative to one's own preferences (e.g., towel recycling). We expect that periodically reminding health care providers of their own prescribing behavior, while providing both a descriptive social norm (displaying the behavior of the best performing peers in their region) and an injunctive norm (citing the national recommended guidelines) will lead providers to conform more closely to these norms. A seminal study by Kiefe et al. demonstrated that providers who were shown their own performance in relation to 90th percentile performance on measures of preventive and chronic disease care had greater performance

improvements than those who were shown their own performance in relation to mean performance.[14] Thus, in the current study those in the Peer Comparison conditions will be provided personalized feedback along with the antibiotic over-prescribing rate of only the top performers within their clinic. In addition, injunctive norms (i.e., indicators of socially desirable performance for high performers) are often excluded. These factors suggest that the use of benchmarks can be improved by applying "nudging" interventions with foundations in social decision making. Our performance feedback reports for each provider randomized to receive the Peer Comparison intervention will have three key characteristics: (1) each target provider will receive his or her individual performance, (2) benchmarks will prominently feature the performance of providers who would be considered credibly peers of the target provider, and (3) benchmarks will reflect *only* performance that is desirable (e.g., showing only the performance of the best-performing credible peers).

3. STUDY DESIGN

The Use of Behavioral Economics and Social Psychology to Improve Treatment of Acute Respiratory Infections (BEARI) Trial is a multisite, cluster-randomized controlled trial with practice as the unit of randomization. The primary aim is to test the ability of three interventions based on behavioral economic principles to reduce the rate of inappropriate antibiotic prescribing for ARIs. We will randomize practices in a 2 x 2 x 2 factorial design to receive up to three interventions for non-antibiotic-appropriate diagnoses: 1) Accountable Justifications: When prescribing an antibiotic for an ARI, clinicians are prompted to record an explicit justification that appears in the patient electronic health record ; 2) Suggested Alternatives: Through computerized clinical decision support, clinicians prescribing an antibiotic for an ARI receive a list of non-antibiotic treatment choices (including prescription options) prior to completing the antibiotic prescription; and 3) Peer Comparison: Each provider's rate of inappropriate antibiotic prescribing relative to top-performing peers will be reported back to the provider periodically by email. We will enroll approximately 300 clinicians (practicing attending physicians or advanced practice nurses) from 49 participating outpatient clinic sites and collect baseline data. All participating clinicians will receive a brief educational module reviewing ARI treatment guidelines at the time of consent and enrollment. Surveys will be administered at the time of enrollment and after the intervention is complete. The primary outcome is the antibiotic prescribing rate for office visits with non-antibiotic-appropriate ARI diagnoses. Secondary outcomes will examine antibiotic prescribing more broadly. The 18- month intervention period will be followed by a one year follow-up period to measure persistence of effects after interventions cease. Data from electronic medical records for participating practices are transferred to the Data Coordinating Center on a weekly basis.

4. SELECTION AND ENROLLMENT OF PARTICIPANTS

4.1 Inclusion Criteria

The subjects involved in this trial are clinicians who will be recruited from multiple clinical sites in Boston and Los Angeles. The target group of physicians (and the patients that they treat) is fully inclusive and representative. Clinicians will be eligible if they treat adult patients with acute respiratory infections. All consenting clinicians at these practices will be offered enrollment.

Each study clinic is required to have an electronic health record (EHR) system in place and have its own physical building (as opposed to multiple clinics sharing the same space, such as the floor of a hospital, where interactions between providers assigned to different intervention groups would be more likely). Clinicians must meet the following inclusion criteria to participate in this study: 1) treat adult patients with acute respiratory infections and practice at one of the study clinics.

An office visit is eligible for inclusion in the outcome denominator if: 1) the patient was 18 years old or older, 2) the provider and practice site were enrolled in the study, and 3) the visit occurred during the 18-month intervention period.

4.2 Exclusion Criteria

Visits will be excluded from the primary analysis when: 1) patients have certain medical co-morbidities that make ARI guidelines less likely to apply, 2) patients have concomitant visit diagnoses indicating a non-ARI possible bacterial infection, 3) patients have concomitant visit diagnoses indicating potentially antibiotic appropriate ARI diagnoses or other ARI diagnoses suggestive of a bacterial infection or 4) the visit occurred within 30 days of an earlier ARI diagnosis. Visits for which a provider records another condition that is not an ARI for which antibiotics might be indicated will also be excluded from the analysis. The sets of diagnoses which will be used to calculate the outcomes are listed in Appendix E: Code Set Definitions.

4.3 Study Enrollment Procedures

All clinicians with adult patients in participating practices will be contacted by email and in-person meetings. Enrollment and consent will be conducted using an online survey administration application.
The email includes a description of the broad goals of the study, a general description of the intervention, compensation providers would receive for participation, and a link to the electronic consent form and baseline survey.
The baseline survey includes an educational module. After providing consent, providers are asked to complete a 15 to 20 minute online survey and educational module. The educational module contains information about ARIs derived from evidence based guidelines and systematic reviews. The educational module also describes the interventions to which a clinician's site was assigned,

including changes they would observe in their EHR (for Accountable Justifications and Suggested Alternatives interventions) and examples of the kinds of emails they would receive (Peer Comparison). These examples include Appendix B: Example of Suggested Alternatives Order Set and Appendix C: Sample Peer Comparison Email Text.

5. STUDY INTERVENTIONS

5.1 Interventions, Administration, and Duration

The intervention period will be 18-months in length for all participants, with a one year follow-up period to measure persistence of effects after interventions end. The pre-intervention baseline period will be 6 months in length.

5.2 Handling of Study Interventions

The following interventions will be compared: *Accountable Justification (AJ)* is an EHR-based intervention that will prompt the clinician to justify, in a free text response, the decision to prescribe an antibiotic for each ARI. The prompt is designed to inform the clinician that the justification will be seen by others in the patient's medical record as an "Antibiotic Justification" note, and that if no justification is entered, the phrase "no justification given" will appear in the note. *Suggested Alternatives (SA)* is an EHR-based intervention most closely resembling traditional clinical decision support alerts and order sets. ARI diagnoses will trigger a pop-up screen that states, "Antibiotics are not generally indicated for [this diagnosis]. Please consider the following prescriptions, treatments, and materials to help your patient." The screen will then suggest a list of alternatives see Appendix D: Example of Suggested Alternatives order set), each with a streamlined order entry option, such as for over-the-counter and prescription medications (e.g., decongestants) and letter templates excusing patients from work. *Peer Comparison (PC)* is an email-based intervention. Clinicians will be ranked from highest to lowest inappropriate prescribing rate within each region using EHR data. Clinicians with the lowest inappropriate prescribing rates (the top-performing 10th percentile) will be informed that they are a "Top Performer" in a congratulatory email. The remaining clinicians will be told that they are "Not a Top Performer" by email. Emails will include the number and proportion of inappropriate antibiotic prescriptions written for a month for non-antibiotic-appropriate ARI cases and the proportion written by Top Performers.

5.3 Adherence Assessment

In order to ensure that the study interventions are being reliably delivered we will create testing scripts that cover logical and coding variation in EHR-based interventions. Study staff will conduct site visits regularly during the intervention to ensure that tests do not fail.

Throughout the course of the study, we will also be monitoring "diagnostic drift" that may result in provider shifting diagnosis to avoid guideline conflicts that might trigger alerts or poor performance reports. Auditing programs that measure diagnostic deviation from each clinician's 2009-2010 rates of ARI ICD-9s will trigger alerts sent to clinic coordinators if there is a statistically significant increase or decrease in the proportion of encounters coded as likely bacterial ARIs vs. likely viral ARIs. If diagnostic drift is detected, an email will be sent to the clinician indicating that the study team has observed discrepancies and that study participation requires accurate diagnoses. If three such emails are sent without evidence that a provider has corrected the pattern, s/he will be eliminated from the study.

6. STUDY PROCEDURES

6.1 Schedule of Evaluations

Assessment	Screening: Baseline prescribing (Month -17 to Month 0)	Baseline, Enrollment, Randomization: (Day 1)	Intervention start (Month 1)	Continuously Measured or monitored	Intervention end: (Month 18)	Follow-up period: (Month 19 to Month 30)
**Clinician-level Assessments**						
Informed Consent Form		X				
Demographics		X				
Inclusion/Exclusion Criteria	X	X				
Provider Attitudes Survey		X			X	
**Visit-level assessments**						
ICD-9 codes	X	X	X	X	X	X
Ordering Data	X	X	X	X	X	X
Adverse Events			X	X	X	

6.2 **Description of Evaluations**

6.2.1 Screening Evaluation

Consenting Procedure

With the assistance of each site's medical director, we will send providers at participating sites an introductory email that includes a description of the broad goals of the study, a general description of the intervention, and a link to the electronic consent form and baseline survey. The consent document will indicate that participation is voluntary and that decisions to participate (or not) will have no bearing on any provider's status at his or her clinic. Providers who provide consent to participate will be asked to complete an online survey and brief educational session prior to the intervention phase, permit de-identified patient records pertaining to patients who saw them for ARIs to be included in the study database, and complete a 15 minute post-intervention survey. We will also describe compensation that providers will receive for participation. We will send up to 6 follow up emails to providers who do not respond, and study personnel will contact them in person when feasible.

6.2.2 Enrollment, Baseline, and/or Randomization

Enrollment

Enrollment date will be documented on the online consent form at the time of consent. Interventions will be initiated after all clinicians in a practice have been enrolled or declined to participate.

Baseline Assessments

- Baseline prescribing rates
- Baseline survey to assess provider characteristics and provider attitudes toward practice guidelines, clinical decision support, electronic health records, and practice environment.

Randomization

We will implement a cluster-randomized design at the clinic level to avoid contamination that might occur if individual providers in close proximity are randomized to different interventions. Providers who practice at multiple clinics will be assigned to the intervention of the clinic for which they spend at least 85% of their time. Geographically distinct individual clinics will be the unit of randomization. We will conduct a block randomization of clinics by clinic organization.[15]

7. SAFETY ASSESSMENTS

Data for patients who have a return visit to a study clinic within 30 days of an eligible study visit with a diagnosis that could represent a serious complication of an untreated bacterial infection (e.g. acute rheumatic fever, head and neck abscess, intracranial abscess, Lemierre syndrome,

mastoiditis, meningitis, pneumonia, sepsis, etc.) will be extracted from study site EHRs and reported to the Data Safety and Monitoring Board.

7.1 Specification of Safety Parameters

Data elements from qualifying ARI visits for providers enrolled in the study will be collected from the electronic health record. At Partners Healthcare, total qualifying visits will be based on qualifying ICD-9 codes (See Appendix E: Code Set Definitions), while Los Angeles sites also incorporate exclusions of suppressor codes used in the decision to trigger the clinical decision support. Aggregate counts of total ARI visits across sites for which the intervention was triggered, including those for which an antibiotic was not prescribed, and the number of return visits occurring within 30 days of index visits (and associated rate of return) with a diagnosis of concern (See Appendix E for diagnoses of concern at revisit) will be calculated. Of return visits identified with a diagnosis of concern, a random sample of 20% of cases will be generated for chart review by site physicians to determine the possibility of study interventions interfering with proper diagnosis and treatment of a patient. Cases will be examined to determine whether earlier antibiotics would have improved the outcomes and classified as one of the following: earlier antibiotics UNLIKELY to have improved the course, early antibiotics MAY HAVE improved the course, UNCERTAIN if earlier antibiotics would have improved the course.

7.2 Methods and Timing for Assessing, Recording, and Analyzing Safety Parameters

Annual reports of our safety measures will be delivered to our Data Safety Monitoring Board.

7.3 Adverse Events

Adverse events are defined as the number of return visits occurring within 30 days of index visits (and associated rate of return) with a diagnosis of concern (See Appendix E for diagnoses of concern at revisit).

7.4 Reporting Procedures

The Principal Investigator will report any unanticipated events to the IRB as well as the Data Safety and Monitoring Board (DSMB) assembled for this study. When notified of an unanticipated event, the DSMB will convene and make a decision as to whether the study should continue. The IRB will also be notified of the DSMB's decision.

7.5 Safety Monitoring

A Data Safety and Monitoring Board (DSMB) has been established. The board is composed of three physician experts in acute respiratory infection care, and will meet biannually throughout the duration of the study to review patient safety and adverse events. This board is composed of 3 members both within and outside the University: 1) Stanley Azen, PhD, Assistant Dean for Research Integrity; Co-Director, Division of Biostatistics; Professor of Preventive Medicine, USC Keck School of Medicine, 2) Rowena J. Dolor, MD, MHS, Associate Professor, Division of General Internal Medicine Director, Duke Primary Care Research Consortium Associate Director, Duke Evidence-based Practice Center, Duke Clinical Research Institute and 3) James

W. Mold, MD, MPH Director of Research, University of Oklahoma Health Sciences Center College of Medicine Dept. of Family and Preventative Medicine.

8. INTERVENTION DISCONTINUATION

Following each DSMB meeting, the board will make recommendations to the local IRBs as to whether the study should continue or if changes to the protocol are necessary for continuation.

9. STATISTICAL CONSIDERATIONS

9.1 General Design Issues

Hypotheses

Our primary hypothesis is that practices randomized to receive behavioral economic interventions will have decreases over time in antibiotic prescribing rates for non-antibiotic appropriate ARIs, compared to contemporaneous antibiotic prescribing rates for non-antibiotic appropriate ARIs among control practices. This hypothesis will be evaluated in an intent-to-treat difference-in-differences framework using a mixed-effects logistic regression model. Fixed effects will include the effects of interventions over time (i.e., interactions between randomization assignment and time), using a 6-months prior to the intervention baseline period. Providers and randomization unit (clinic) will be modeled as random effects.

Design

We will conduct a between-group factorial cluster randomized trial of ambulatory clinic visits in a national sample of clinics. Clustering (by clinic) helps us prevent treatment contamination between individual clinicians within the same clinic. The factorial design will allow us to study the effects of multiple antibiotic policies as often happens in the real-world, where State and Federal public health as well as clinic organization quality improvement interventions may be happening at the same time. Using this factorial design, three interventions will be tested for their ability to alter inappropriate physician prescribing behavior: 1) Accountable Justifications triggers by guideline-discordant prescriptions that ask providers to provide their rationale for prescribing an antibiotic and include these rationales in the medical record; 2) Suggested Alternatives presents in EHR order sets containing guideline concordant treatment options for ARIs; and 3) Peer Comparisons communicates through emailed performance feedback reports that compare each provider's own performance to his or her top-performing peers.

Outcome measures

The primary outcome measure is the rate of antibiotic prescribing for non-antibiotic-appropriate acute respiratory infections.

The ICD-9 codes for primary outcomes are defined in detail in Sections 9.4.1 and 9.4.2 of

this protocol document. These outcomes are computable clinical quality measures from the electronic health record. These are widely used in medicine to evaluate quality improvement and reliability and validity are generally supported.[16] As a secondary outcome, effects on potentially appropriate acute respiratory infection diagnosis will be evaluated with respect to diagnostic drift and safety (see Section 9.4.2).

An office visit is eligible for inclusion in the outcome denominator if: 1) the patient was 18 years old or older, 2) the provider and practice site were enrolled in the study, 3) the visit occurred during the 18- month intervention period, and 4) the patient did not have a visit with any ARI diagnosis in the prior 30 days. Visits are excluded from the primary analysis when: 1) patients have certain medical co-morbidities that make ARI guidelines less likely to apply, 2) patients had concomitant visit diagnoses indicating a non-ARI possible bacterial infection, or 3) patients had concomitant visit diagnoses indicating potentially antibiotic appropriate ARI diagnoses or other ARI diagnoses suggestive of a bacterial infection. Visits for which a provider recorded another condition that was not an ARI for which antibiotics might be indicated were also excluded from the analysis.

9.2 Sample Size and Randomization

Using the correction for inter-cluster correlation from Kish,[17] we estimated the power of our study to detect a clinically significant difference between binary conditions. That is, the sample size must be inflated by a factor of $1+\theta(m\text{-}1)$, where θ is the inter class correlation and m is the number of ARI observations per cluster. In our calculations we assumed an intra-clinic correlation of 0.05 and assumed a baseline antibiotic prescribing rate of 50%, an ARI visit rate of 15 visits per month for full time providers, and independence of treatments in the factorial design. We calculated the number of visits needed for an 80% chance (1 – Type II error) to detect a clinically meaningful difference in antibiotic prescribing (7%). We assumed Type I alpha is equal to 0.05, a 75% recruitment success rate for recruiting 376 eligible providers across 49 sites, resulting in 141 providers per study factor (282 clinicians total) and a one-sided α of 0.05. To achieve statistical power of 0.80 would require a total of 2,252 visits at each factor level, or 4,504 visits across all study conditions. Therefore, if each provider had a minimum of 16 antibiotic-inappropriate ARI visits over the course of the study, we would have sufficient power to detect a clinically significant effect. Randomization is described next in Section 9.2.1.

9.2.1 Treatment Assignment Procedures

Randomization of study sites

We have chosen a cluster-randomized design at the clinic level to avoid contamination that might occur if individual providers in close proximity are randomized to different interventions. Providers who practice at multiple clinics are assigned to the intervention of the clinic for which they spend at least 85% of their time.

Geographically distinct individual clinics will be treated as the unit of randomization. These are clinics belonging to one of three larger clinical organizations covering a connected

geographic area in either Massachusetts (Partners Healthcare consisting of Brigham and Women's Hospital and Massachusetts General Hospital affiliated primary care practices) or Southern California (AltaMed; The Children's Clinic). We will carry out a block randomization of clinics by clinic organization using the statistical computing language R. We first will construct two matrices that each represent a main effects design and together represented the full factorial design (2 x 2 x 2 design). For each clinic organization, we will construct ordered collections of clinics. We then will employ the sample function in R to return a random permutation of each ordered collection. For each collection of clinic organizations we will draw a sample that represents the largest number of clinics within each clinic organization that was divisible by 8, the number of study arms. We then will use the list function, a function that ties together related data that do not share the same structure, to assign each randomly permutated clinic to a study arm, repeating this process until clinics have filled the eight arms of the study in equal measure. Because the number of clinics at each organization is not always divisible by eight, we will treat "remainder" clinics across all organizations differently. These remainder clinics will be randomized to conditions within one of the fractional factorial main effects design (a subset of the larger 2 x 2 x 2 design) to maximize power for main effects estimates. This will be accomplished in a procedure similar to the one described above. One of the two possible fractional factorial designs of the larger 2 x 2 x 2 design will be chosen randomly to assign remainder clinics to a condition so that remainder clinics have an ex ante equal probability of assignment to any one of the eight conditions in the full factorial design. Allocation of the sequence will be concealed until after the interventions were assigned.

9.3 Interim analyses and Stopping Rules

No interim analysis will be conducted on primary or secondary outcomes. The Data Safety and Monitoring board is granted the power to recommend discontinuation of the study to each study IRB, if safety concerns are found. The board will meet biannually throughout the duration of the study to review patient safety and adverse events. Following each meeting, the board will make recommendations to the local IRBs as to whether the study should continue or if changes to the protocol are needed. Data for patients who have a return visit to a study clinic within 30 days of an eligible study visit with a diagnosis that could represent a serious complication of an untreated bacterial infection (e.g. acute rheumatic fever, head and neck abscess, intracranial abscess, Lemierre syndrome, mastoiditis, meningitis, pneumonia, sepsis, etc.) will be extracted from study site EHRs and reported to the Board.

9.4 Outcomes

9.4.1 Primary outcome

The primary outcome is defined as the antibiotic prescribing rate for acute respiratory infection diagnoses changes in antibiotic prescribing rate for the following ICD-9 diagnoses: 460 Acute nasopharyngitis (common cold); 464 acute laryngitis and tracheitis;

465 Acute laryngeopharyngitis/acute upper respiratory infection; 466 Acute bronchitis; 490 Bronchitis not specified as acute or chronic; and 487 Influenza.

9.4.2 Secondary outcomes

To study safety and diagnostic drift we will evaluate an expanded list of potentially appropriate and other diagnoses of interest. For potentially antibiotic appropriate acute respiratory infection diagnoses these are: Acute sinusitis; Acute sinusitis/rhinosinusitis; Acute pharyngitis; 462 Acute pharyngitis. For other acute respiratory infection diagnoses or symptoms of interest these are: Streptococcal sore throat; 034.0 Acute pharyngitis; Cough; and 786.2 Acute bronchitis.

9.5 Data Analyses

We will use the following descriptive statistics to characterize the sample: Means and medians for continuous measures, frequencies for count data, standard deviations and interquartile ranges for variance.

For inferential analysis of our hypotheses, we will employ a mixed-effects hierarchical logistic regression model to estimate the adjusted marginal effect over time of each intervention on the primary outcome using. Fixed effects will include intervention assignment, time period dummy variables (the baseline prescribing rate for each clinician, the intervention and intervention months 0-6, 7-12, and 13-18), and time period interacted with intervention assignment. Providers and randomization unit (clinic) will be included as random effects. To isolate the effect of each individual intervention (Suggested Alternatives, Accountable Justification, or Peer Comparison) on the primary outcome, controlling for any co-occurring interventions under the factorial design.

To assess diagnostic drift, we will use the same analytic model as for the primary outcome, but with the percentage of *all* ARIs that is coded as antibiotic-appropriate in each study arm as the dependent variable (a secondary outcome).

10. DATA COLLECTION AND QUALITY ASSURANCE

10.1 Data Collection Forms

Two types of data will be collected – data from electronic medical and billing records and data from self-administered online surveys at the beginning and end of the study.

10.2 Data Management

Each of the participating sites will create an extract from their Electronic Medical or Billing Records of the Data Elements for all patients with an upper respiratory infection. These records will be transferred to the coordinating center on a weekly basis.

The CC has created programs and quality control queries for transforming all of the data into a standard model (Observational Medical Outcomes Partnership Common Data Model, version 3).

The data collection forms will be online surveys. Each of the electronic data systems, Epic, NextGen, and the Longitudinal Medical Record and Partners Healthcare billing system will have native data capture formats.

10.3 Quality Assurance

10.3.1 Training

Staff will be trained on the permissible values present in Electronic Records, frequency of update, and expected volumes of data.

10.3.2 Quality Control Committee

The quality control committee will consist of practicing clinicians from each participating clinical organization. They will review automatically refreshing dashboards for potential deviations in coding systems and appropriate values for codes for inclusion in the outcome measures. These dashboards will be reviewed prior to each email distribution.

10.3.3 Metrics

Quality control metrics will be based on reports verifying visits with ARI ICD-9 Codes. All drugs prescribed at these visits will be categorized as “antibiotic” or “non-antibiotic”. Incorrect categorizations will be corrected and outcome computations recomputed before each email is delivered.

10.3.4 Protocol Deviations

Our task tracking system, JIRA will be used to track and document issues. Each issue will include both an assignee and a reviewer.

10.3.5 Monitoring

In addition to data quality reviews, we will also review the integrity of the interventions.

On an approximately quarterly basis, staff will visit headquarters of participating sites and verify functionality of decision support tools.

Additionally, practicing clinicians on our study team will have the ability to monitor electronic medical record interventions in their own health systems.

11. PARTICIPANT RIGHTS AND CONFIDENTIALITY

11.1 Institutional Review Board (IRB) Review

The study protocol and the informed consent document for all clinic sites will be reviewed and approved by the University of Southern California’s Institutional Review Board (IRB).

Individual site protocols will also be submitted for review and approval by the Massachusetts and California site local IRBs.

11.2 Informed Consent Forms

An electronically signed consent form will be obtained from each participating provider. The consent form will describe the purpose of the study, the procedures to be followed, the risks and benefits of participation, and compensation for participation.

11.3 Participant Confidentiality

Data will be recorded with SSL protected web sites to a data warehouse, and transferred over secure network protocol. Data will be kept in encrypted files on computers in locked offices at USC Schaeffer Center facilities. Only study investigators will have access to a list of study ID codes that will be traceable back to actual subject contact identifiers for clinicians. These codes will be kept in locked offices at USC Schaeffer Center facilities.

Identified data will only be released to providers participating in the "Peer Comparison" arm of the study. As stored, data will be de-identified with MD5 hash to link a participant number (unique to physician) to a primary outcome. The participant number is programmatically re-identified only at the moment of an automatically scheduled e-mail. Study researchers will have password protected access to coded data only.

11.4 Study Discontinuation

Following each DSMB meeting, the board will make recommendations to the local IRBs as to whether the study should continue or if changes to the protocol are necessary for continuation.

12. COMMITTEES

Data Safety Monitoring Board: Stanley Azen, PhD, USC Keck School of Medicine, Rowena J. Dolor, MD, MHS, Duke Clinical Research Institute, James W. Mold, MD, MPH, University of Oklahoma Health, Sciences Center College of Medicine

13. PUBLICATION OF RESEARCH FINDINGS

Publication of results from our research will follow the NIH Public Access Policy, which requires that we submit to the National Library of Medicine's PubMed Central an electronic version of final, peer-reviewed manuscripts upon acceptance for publication, to be made publicly available no later than 12 months after the official date of publication.

14. REFERENCES

1. Grijalva C, Nuorti J, Griffin M. Antibiotic prescription rates for acute respiratory tract

infections in US ambulatory settings. *JAMA.* 2009;302(7):758-766.
2. Gonzales R, Malone D, Maselli J, Sande M. Excessive antibiotic use for acute respiratory infections in the United States. *Clin Infect Dis.* 2001;33:757-762.
3. Shehab N, Patel P, Srinivasan A, Budnitz D. Emergency department visits for antibiotic-associated adverse events. *Clin Infect Dis.* 2008;47(735-743).
4. Arias C, Murray B. Antibiotic-resistant bugs in the 21st century—a clinical super-challenge. *N Engl J Med.* 2009;360:439-443.
5. Klevens R, Morrison M, Nadle J, al e. Invasive methicillin-resistant Staphylococcus aureus infections in the United States. *JAMA.* 2007;298:1763-1771.
6. Lerner J, Tetlock P. Accounting for the effects of accountability. *Psych Bull.* 1999;125(2):255.
7. Johnson EJ, & Goldstein, D. G. (2003). Do defaults save lives? Science, 302, 1338-1339.
8. Madrian B, & Shea, D. (2001). The power of suggestion. Quarterly Journal of Economics, 18-116.
9. Thaler R, Sunstein C. *Nudge: Improving decisions about health, wealth, and happiness.* Yale University Press.; 2008.
10. Macfarlane J, Holmes, W., Macfarlane, R., & Britten, N. (1997). Influence of patients' expectations on antibiotic management of acute lower respiratory tract illness in general practice: Questionnaire study. British Medical Journal, 315, 1211-1214.
11. Cialdini R, Reno R, Kallgren C. A focus theory of normative conduct: recycling the concept of norms to reduce littering in public places. *J Pers Soc Psych.* 1990;58(6):1015.
12. Cialdini RB. *Influence: Science and Practice. 5th Edition.* Boston: Allyn & Bacon; 2010.
13. Goldstein N, Cialdini R, Griskevicius V. A room with a viewpoint: Using social norms to motivate environmental conservation in hotels. *J Consum Res.* 2008;35:472-482.
14. Kiefe C, Allison J, Williams O, Person S, Weaver M, Weissman N. Improving quality improvement using achievable benchmarks for physician feedback: a randomized controlled trial. *JAMA.* 2001;285(22):2871-2879.
15. Ihaka R GR. A language for data analysis and graphics. *J Comput Graph Stat* 1996;5:299-314.
16. Chan KS, Fowles, J. B., & Weiner, J. P. (2010). Electronic health records and reliability and validity of quality measures: a review of the literature.Medical Care Research and Review.
17. Kish L. *Survey Sampling.* New York, NY: John Wiley and Sons; 1965.

15. SUPPLEMENTS/APPENDICES

TABLE 1. ACUTE RESPIRATORY INFECTION DIAGNOSES RELATED TO INTERVENTIONS AND OUTCOMES ASSESSMENTS

Diagnoses		ICD9-CM	Used to Trigger Decision Support
**Non-Antibiotic Appropriate ARI Diagnoses (Included in Peer Comparison and Primary Outcome Assessment)**			
	Acute nasopharyngitis (common cold)	460	non-specific URI
	Acute laryngitis and tracheitis	464, 464.0, 464.00, 464.1, 464.10, 464.2, 464.20, 464.4, 464.50	non-specific URI
	Acute laryngeopharyngitis/acute upper resp infection	465, 465.0, 465.8, 465.9	non-specific URI
	Acute bronchitis	466, 466.0, 466.1, 466.11, 466.19	acute bronchitis
	Bronchitis not specified as acute or chronic	490	acute bronchitis
	Influenza	487, 487.1, 487.8	influenza
**Potentially Antibiotic Appropriate ARI Diagnoses (Included in Secondary Outcome Assessment)**			
	Acute sinusitis	461.xx	acute sinusitis/rhinosinusitis
	Acute pharyngitis	462	acute pharyngitis
**Other ARIs Diagnoses or Symptoms of Interest (Included in Secondary Outcome Assessment)***			
	Streptococal sore throat	034.0	acute pharyngitis
	Cough	786.2	acute bronchitis

Only additional diagnoses triggering clinical decision support are included here. Additional diagnoses included in the secondary outcomes are listed in Supplemental Appendix E.

Appendix A: Survey and sample educational module at start of study

Online Survey

THE ONLINE SURVEY IS INTENDED TO (1) ELICIT INFORMATION FROM PROVIDERS (2) MONITOR IF "EDUCATON" INFLUENCES RESPONSES TO QUESTIONS ABOUT TREATMENT PREFERENCES. RESPONDENTS WILL HAVE THE OPPORTUNITY TO CHANGE THEIR 'FINAL' ANSWERS AT ANY TIME IN THE SURVEY. WE WILL RECORD ALL ANSWERS AND LOG CLICKS ON INFORMATIONAL LINKS PROVIDED.

<u>Basic information about your clinical background.</u>

1. When did you start working at [*name of clinic*]? (<1 year ago, 1-2 years ago, 3-5 years ago, 5-10 years ago, >10 years ago)

2. When did you finish your clinical training as a physician (i.e., your internship, residency, or fellowship—the one you most recently completed)? (<2 years ago, 2-5 years ago, 5-10 years ago, 10-20 years ago, >20 years ago)

3. What is your clinical specialty? (internal medicine, family practice, general practice, pediatrics, other)

<u>Information about the electronic health record (EHR) used at your clinic.</u>

4. How would you rate your overall level of satisfaction with the electronic health record (EHR) used at your clinic?
   (1= Very unsatisfied , 5=Very satisfied)

5. Thinking about your workflow <u>during</u> an office visit with a patient, how often do you enter at least 1 diagnosis for the visit into the EHR <u>while you are still seeing the patient</u>?

   a. Always
   b. Usually
   c. Sometimes
   d. Rarely
   e. Never
   f. Not applicable: The EHR does not offer a way to enter a diagnosis (or diagnoses) that correspond to the visit.

Quality improvement efforts.

6. Within the past year, have you received any feedback—positive or negative—from your clinic about the quality of care you provide to patients (for any kind of care)?

    a. Yes, positive feedback only
    b. Yes, both positive and negative feedback
    c. Yes, negative feedback only
    d. No, did not receive any feedback at all
    e. Unsure / Can't Remember

7. [If yes to previous] Based on the feedback you received, did you make any changes to the way you deliver medical care?

    a. Yes, made 1 or more changes
    b. No, made no changes
    c. Unsure / Can't Remember

8. In the past year, did you attend any medical educational sessions? *Note: "Medical education sessions" include sessions that yielded credit towards maintenance of certification (e.g., CME) and less formal sessions that did not yield such credit.*

    a. Yes
    b. No
    c. Unsure / Can't Remember

9. [If yes to question 8] Based on the information you received in any of these educational sessions, did you make any changes to the way you deliver medical care?

    a. Yes, made 1 or more changes
    b. No, made no changes
    c. Unsure / Can't Remember

10. [If yes to question 8] Did any of the educational sessions you attended cover the office-based treatment of acute respiratory infections (e.g., viral URIs, pharyngitis, bronchitis)?

    a. Yes
    b. No
    c. Unsure / Can't Remember

11. [If yes to question 8] Did any of the educational sessions you attended cover the office-based treatment of acute low back pain?

a. Yes
b. No
c. Unsure / Can't Remember

12. Based on your general experience as a clinician, please indicate how much you agree or disagree with the following statements:

i. Continuing education is an effective way to improve the quality of care (1 = Strongly agree, 2 = Agree, 3 = Neither Agree nor Disagree, 4 = Disagree, 5 = Strongly Disagree)
ii. Auditing physicians' clinical performance and providing performance feedback is an effective way to improve the quality of care (1 = Strongly agree, 2 = Agree, 3 = Neither Agree nor Disagree, 4 = Disagree, 5 = Strongly Disagree)
iii. Electronic decision support tools (e.g., "pop up" reminders in your EHR) are an effective way to improve the quality of care (1 = Strongly agree, 2 = Agree, 3 = Neither Agree nor Disagree, 4 = Disagree, 5 = Strongly Disagree)
iv. Condition-specific, streamlined electronic order sets are an effective way to improve the quality of care (1 = Strongly agree, 2 = Agree, 3 = Neither Agree nor Disagree, 4 = Disagree, 5 = Strongly Disagree)

Your assessment of clinical guidelines.

13. Please indicate your level of knowledge about the following clinical guidelines.	[Know this guideline in detail / Know this guideline in general, but not every detail / Not familiar with this guideline]
Screening	
Guidelines for colorectal cancer screening (USPSTF guideline: http://www.uspreventiveservicestaskforce.org/uspstf/uspscolo.htm)	
Guidelines for breast cancer screening (USPSTF guideline: http://www.uspreventiveservicestaskforce.org/uspstf/uspsbrca.htm)	
Guidelines for cervical cancer screening (USPSTF guideline: http://www.uspreventiveservicestaskforce.org/uspstf/uspscerv.htm)	
Chronic disease care	
Guidelines for the care of diabetes mellitus (ADA guideline: http://www.guideline.gov/content.aspx?id=15687)	
Guidelines for lipid and cholesterol management (ATP III guidelines: http://circ.ahajournals.org/cgi/reprint/106/25/3143)	
Acute care	
Guidelines for antibiotic use in non-specific upper respiratory infections (CDC guidelines: http://www.annals.org/content/134/6/490.abstract)	
Guidelines for imaging in acute low back pain (ACP/APS guidelines: http://www.annals.org/content/147/7/478.full.pdf+html)	

a. **In the grid below, please estimate the AVERAGE time <u>allocated</u> to you and amount of time you feel would be <u>needed</u> to provide high quality care for your patients.** *(please check one box)*

Visit type	Time *allocated*	Time *needed*
i. Complete Physical/Consultation	_______minutes	_______minutes
ii. Routine Follow-up Visits	_______minutes	_______minutes
iii. Urgent Care Visits (in general)	_______minutes	_______minutes
iv. Urgent Care Visits for acute respiratory infections	_______minutes	_______minutes

b. **Which best describes the atmosphere in your office?** *(please check one box)*	Calm, orderly		Busy, but reasonabl e		Hectic, chaotic
	☐1	☐2	☐3	☐4	☐5

c. **Please indicate how much you agree or disagree with the following statement.** *(please check one box)*	Strongly disagree	Disagre e	Neither agree nor disagree	Agree	Strongly Agree
Overall, I am satisfied with my current job	☐1	☐2	☐3	☐4	☐5

Educational Module

Key: AJ = Accountable Justifications, PC = Peer Comparison, SA = Suggested Alternatives.

Guidelines for treating non-specific upper respiratory infections (URIs) in adults

- Definition
  - Acute infection in which sinus, pharyngeal, and lower airway symptoms, although frequently present, are not prominent
  - Also known as "the common cold"
- Causes
  - If systemic symptoms (e.g., myalgias, malaise) are prominent: influenza and parainfluenza infection
  - If systemic symptoms are less prominent: rhinoviruses, coronaviruses, adenoviruses, enteroviruses, and respiratory syncytial virus
- Diagnosis
  - Symptoms may include cough, sore throat, runny nose, nasal congestion, headache, low grade fever, facial pressure, sneezing
  - Purulent secretions from nares or throat do NOT indicate the presence of bacterial infection
- Course of illness
  - Duration of symptoms is usually 7-10 days.
- Guideline-consistent treatments
  - *These guidelines apply to immunocompetent adults without complicating comorbid conditions, such as chronic lung or heart disease*
  - Treat with decongestants, cough suppressants, and/or analgesics/antipyretics. For some patients, albuterol may also be appropriate.
  - Patient education: fluids, rest, salt water gargle.
  - Some patients may need a work excuse letter.
  - Antibiotics are <u>not</u> indicated.
  - (<u>CDC recommendation link here</u>)

<u>*Response tasks (for PC-/AJ-/SA- subjects)*</u>

- Thinking about the patients you see in clinic for non-specific URIs, how often does the guideline-based recommendation against prescribing antibiotics apply? [0-20% / 21-40% / 41-60% / 61-80% / 81-100% of patients / DK]
- How frequently do you prescribe antibiotics to your patients for the treatment of non-specific URIs? [0-20% / 21-40% / 41-60% / 61-80% / 81-100% of visits / DK]
- How frequently do you think other clinicians in your practice prescribe antibiotics for the treatment of non-specific URIs? [0-20% / 21-40% / 41-60% / 61-80% / 81-100% of visits/ DK]
- In some online studies, a small number of participants do not pay close attention to all of the items they are answering. To indicate that you are paying close attention, please do not mark any of the choices for the following question: How frequently do you think physicians prescribe decongestants for non-specific URIs? [0-20% / 21-40% / 41-60% / 61-80% / 81-100% of visits / DK]"

<u>*Additional statements (for PC+ subjects)*</u>

[Injunctive norm] These guidelines for treating non-specific URIs have been endorsed by the American Academy of Family Physicians, the American College of Physicians, the Infectious Diseases Society of America, and the Centers for Disease Control and Prevention.

Response task additions (for PC+ subjects)

- None (same as PC-)

Intervention summary (for PC+ subjects)

- During the study, you will receive regular updates on your own rate of antibiotic prescribing for patients who have non-specific URIs. As a demonstration of achievable performance, these updates will also include the antibiotic prescribing rate achieved by the 10% of physicians in [name of clinic] whose prescribing is most guideline-concordant.

Additional statements (for AJ+ subjects)

[Importance of justification] Guidelines are intended to help clinicians treat the majority of their patients. However, there can be clinical reasons why a guideline might not apply to a particular patient. When there is a good clinical reason, a physician might justifiably choose not to follow a guideline.

If a physician decides that there is a clinically justifiable reason to prescribe antibiotics to a patient with a non-specific URI, he or she should have a clear sense of what this reason is. He or she should be able to state the justification for not following the guideline.

Response task additions (for AJ+ subjects)

- To what extent do you agree with these overall guidelines for treating non-specific URIs? [completely agree to completely disagree]
- To what extent do you agree that antibiotics are not indicated for the treatment of non-specific URIs? [completely agree to completely disagree]
- (Other response tasks same as AJ-)…

Intervention summary (for AJ+ subjects)

- During the study, if you prescribe antibiotics to patient who you are seeing for a non-specific URI, you will be asked to supply a brief written justification for prescribing antibiotics. The justification that you write will be entered in the patient's medical record. If you do not write a justification, the phrase "No justification for prescribing antibiotics was given" will appear in your encounter note.

Additional statements (for SA+ subjects)

[Reminder about alternatives to antibiotics] For non-specific URIs, patients most often want a diagnosis and relief from symptoms; only a minority want antibiotics. Instead of antibiotics, you can prescribe medications that treat congestion, cough, sore throat, and general aches and pains to provide symptomatic relief. In most of these categories, there are both over-the-counter (OTC) and prescription options.

Even for OTC medications, writing a prescription can help your patients. In addition to serving as a reminder, writing a prescription will allow your patients to use their Flexible Savings Accounts (FSAs). Without your prescription, patients will be unable to use their FSAs to buy OTC medications.

In addition to medications, you can give patients educational materials that provide information and reassurance. You can use these materials as non-antibiotic treatments that address patients' most common concerns.

Response task additions (for SA+ subjects)

- None (same as SA-)

<u>Intervention summary (for SA+ subjects)</u>

- During the study, when you prescribe a medication to a patient who you diagnose with non-specific URI, you will be shown a list of non-antibiotic alternative prescriptions and symptomatic treatments. You will be able to select from among these treatment options, and corresponding prescriptions will be generated. You will also be able to select patient educational materials that will be printed for the patient you are seeing.
- Here is the list of non-antibiotic treatments that will be offered to you. If you want to prescribe a medication that does not appear on the list (including an antibiotic), you will be able to write this prescription as usual by closing the list. [show SA list below]

Guidelines for treating acute sinusitis/rhinosinusitis in adults

- Definition
  - "Sinusitis" refers to inflammation of the mucosa of the paranasal sinuses. Because inflammation of the nasal mucosa always accompanies sinusitis, "rhinosinusitis" has become the preferred term.
  - Rhinosinusitis is <u>acute</u> when of duration less than 4 weeks
- Causes
  - Most cases of acute rhinosinusitis diagnosed in ambulatory care are caused by uncomplicated viral upper respiratory tract infections.
  - Acute bacterial rhinosinusitis is usually a secondary infection resulting from sinus obstruction or impairment of mucus clearance mechanisms caused by an acute viral upper respiratory tract infection.
- Diagnosis
  - Patients with rhinosinusitis symptoms that last less than 7 days are unlikely to have bacterial infection, although rarely some patients with acute bacterial rhinosinusitis present with dramatic symptoms of severe unilateral maxillary pain, swelling, and fever.
  - The clinical diagnosis of acute <u>bacterial</u> rhinosinusitis should be reserved for patients with rhinosinusitis symptoms lasting 7 days or more <u>and</u> who have maxillary pain or tenderness in the face or teeth (especially when unilateral) and purulent nasal secretions.
- Course of illness
  - Acute rhinosinusitis resolves without antibiotic treatment in most cases.
  - Duration of symptoms is usually 7-10 days, but longer duration alone does <u>not</u> reliably indicate bacterial etiology.
- Guideline-consistent treatments
  - *These guidelines apply to adults who are not immunocompromised*
  - Treat with topical and systemic decongestants and/or analgesics/antipyretics.
  - Sinus radiography is not recommended.
  - Antibiotic therapy should be <u>reserved</u> for patients with moderately severe symptoms who meet the criteria for the clinical diagnosis of acute bacterial rhinosinusitis and for those with severe rhinosinusitis symptoms—especially those with unilateral facial pain—regardless of duration of illness.
    - For initial antibiotic treatment, use the most narrow-spectrum agent active against the likely pathogens, Streptococcus pneumoniae and

Haemophilus influenzae: amoxicillin, doxycycline, and trimethoprim-sulfamethoxazole.

– (CDC recommendation link here)

Response tasks (for PC-/AJ-/SA- subjects)

- Thinking about the patients you see in clinic for acute sinusitis/rhinosinusitis, how often do the guideline-based recommendations for prescribing antibiotics apply? [0-20% / 21-40% / 41-60% / 61-80% / 81-100% of patients / DK]
- How frequently do you prescribe antibiotics to your patients for the treatment of acute sinusitis/rhinosinusitis? [0-20% / 21-40% / 41-60% / 61-80% / 81-100% of visits / DK]
- How frequently do you think other clinicians in your practice prescribe antibiotics for the treatment of acute sinusitis/rhinosinusitis ? [0-20% / 21-40% / 41-60% / 61-80% / 81-100% of visits / DK]

Additional statements (for PC+ subjects)

[Injunctive norm] These guidelines for treating acute sinusitis/rhinosinusitis have been endorsed by the American Academy of Family Physicians, the American College of Physicians, the Infectious Diseases Society of America, and the Centers for Disease Control and Prevention.

Response task addition (for PC+ subjects)

- None (same as PC-)

Intervention summary (for PC+ subjects)

- During the study, you will receive regular updates on your own rate of antibiotic prescribing for patients who have acute sinusitis/rhinosinusitis. As a demonstration of achievable performance, these updates will also include the antibiotic prescribing rate achieved by the 10% of physicians in [name of clinic] whose prescribing rates are lowest.

Additional statements (for PC+ subjects)

[Injunctive norm] These guidelines for treating acute sinusitis/rhinosinusitis have been endorsed by the American Academy of Family Physicians, the American College of Physicians, the Infectious Diseases Society of America, and the Centers for Disease Control and Prevention.

Response task addition (for PC+ subjects)

- None (same as PC-)

Intervention summary (for PC+ subjects)

- During the study, you will receive regular updates on your own rate of antibiotic prescribing for patients who have acute sinusitis/rhinosinusitis. As a demonstration of achievable performance, these updates will also include the antibiotic prescribing rate achieved by the 10% of physicians in [name of clinic] whose prescribing rates are lowest.

Additional statements (for SA+ subjects)

[Reminder about alternatives to antibiotics] Regardless of whether antibiotics are prescribed for acute sinusitis/rhinosinusitis, decongestants may enable drainage of sinus secretions, and analgesics/antipyretics can provide symptomatic relief. You can also prescribe these medications instead of antibiotics when antibiotics are not

indicated. There are both over-the-counter (OTC) and prescription-only options for decongestants and analgesics/antipyretics.

Even for OTC medications, writing a prescription can help your patients. In addition to serving as a reminder, writing a prescription will allow your patients to use their Flexible Savings Accounts (FSAs). Without your prescription, patients will be unable to use their FSAs to buy OTC medications.

In addition to medications, you can give patients educational materials that provide information and reassurance. You can use these materials as non-antibiotic treatments that address patients' most common concerns.

Response task addition (for SA+ subjects)

- None (same as SA-)

Intervention summary (for SA+ subjects)

- During the study, when you prescribe a medication to a patient who might have acute sinusitis/rhinosinusitis, you will be shown a list of non-antibiotic alternative prescriptions and symptomatic treatments. You will be able to select from among these treatment options, and corresponding prescriptions will be generated. You will also be able to select patient educational materials that will be printed for the patient you are seeing.
- Here is the list of non-antibiotic treatments that will be offered to you. If you want to prescribe a medication that does not appear on the list (including an antibiotic), you will be able to write this prescription as usual by closing the list. [show SA list below]

Additional statements (for AJ+ subjects)

[Importance of justification] Guidelines are intended to help clinicians treat the majority of their patients. However, there can be clinical reasons why a guideline might not apply to a particular patient. When there is a good clinical reason, a physician might justifiably choose not to follow a guideline.

For acute sinusitis/rhinosinusitis, antibiotics may be prescribed in guideline-consistent or guideline-inconsistent ways. In either case, a physician who decides that there is a clinically justifiable or guideline-consistent reason to prescribe antibiotics to a patient with acute sinusitis/rhinosinusitis should have a clear sense of what this reason is. He or she should be able to state this justification or explain how the guideline was followed.

Response task additions (for AJ+ subjects)

- To what extent do you agree with these overall guidelines for treating acute sinusitis/rhinosinusitis? [completely agree to completely disagree]
- To what extent do you agree that antibiotics are <u>not</u> indicated for the treatment of acute sinusitis/rhinosinusitis of <7 days' duration in most patients? [completely agree to completely disagree]
- (Other response tasks same as AJ-)…

Intervention summary (for AJ+ subjects)

- During the study, if you prescribe antibiotics to patient who you are seeing for acute sinusitis/rhinosinusitis, you will be asked to supply a brief written justification for prescribing antibiotics. The justification that you write will be entered in the patient's medical record. If you do not write a justification, the phrase "No justification for prescribing antibiotics was given" will appear in your encounter note.

Guidelines for treating acute pharyngitis in adults

- Definition
  - Cases of acute sore throat of <7 days duration in which specific rarer causes of sore throat (e.g., gonococcus, diphtheria,

epiglottitis, Ludwig angina, acute HIV infection, retropharygeal abscess, trauma) are not present.

- Causes
  - Viruses are the most common cause
  - Group A beta-hemolytic streptococcus (GABHS) is the cause of approximately 10% of adult cases of pharyngitis
- Treatment principles
  - The major reason to treat adults with GABHS pharyngitis is symptomatic relief. In patients with GABHS, antibiotic therapy instituted within 2 to 3 days of symptom onset shorten the duration of symptoms by 1 to 2 days.
  - Antibiotics do not hasten symptomatic improvement in patients without GABHS.
  - Complications of GABHS pharyngitis are rare in immunocompetent adults.
    - In cases of GABHS pharyngitis, there is no evidence that antibiotics reduce the incidence of acute glomerulonephritis.
    - In the United States, the incidence of acute rheumatic fever fell by a factor of 60 between 1965 and 1994. In order to prevent one case of acute rheumatic fever, current estimates of the number of patients with GABHS that would need treatment with antibiotics range from 3000-4000 patients.
  - There is no evidence that the use of antibiotics reduces the spread of GABHS among non-institutionalized adult patients. The pre-symptomatic incubation period for GABHS is 2-5 days, during which patients may expose their close contacts.
- Diagnosis
  - The most reliable predictors of GABHS pharyngitis are the Centor criteria:
    - Tonsillar exudates
    - Tender anterior cervical lymphadenopathy or lymphadenitis
    - Absence of cough
    - History of fever
  - The positive predictive value of the presence of 3-4 Centor criteria is 40-60% for GABHS pharyngitis.
  - The negative predictive value of 0, 1, or 2 Centor criteria is ~80% for the absence of GABHS pharyngitis.
  - Rapid antigen tests for GABHS can be combined with the Centor criteria
  - Throat cultures are not recommended for routine use in cases of acute pharyngitis in adults
- Course of illness
  - For viral acute pharyngitis, the duration of symptoms is usually 5 to 7 days.

- Guideline-consistent treatments (for immunocompetent adults without complicated comorbid conditions, such as chronic lung or heart disease, or history of rheumatic fever, in the absence of known local GAHBS outbreaks)
  - Treat all patient with analgesics/antipyretics. For patients with co-occurring nasal congestion and post-nasal drip, decongestants may also be helpful.
  - Patient education: fluids, rest, salt water gargle.
  - Some patients may need a work excuse letter

- Use of antibiotics: three strategies are acceptable
    1. Test patients (rapid antigen) with 2, 3, or 4 Centor criteria, and limit antibiotic therapy to patients with positive test results.
    2. Test patients (rapid antigen) with 2 or 3 Centor criteria test, and limit antibiotic therapy to patients with positive test results or patients with four criteria.
    3. Do not use any diagnostic tests, and limit antibiotic therapy to patients with 3 or 4 Centor criteria.
- If antibiotics are used, guideline-consistent options are:
    1. a single dose of intramuscular penicillin G benzathine (1.2 MU for adults)
    2. standard penicillin VK, 500 mg orally twice or three times daily for 10 days
    3. in penicillin-allergic patients, use erythromycin 500 mg twice daily
- (CDC recommendation link here)

Response tasks (for PC-/AJ-/SA- subjects)

- Thinking about the patients you see in clinic for acute pharyngitis, how often do the guideline-based recommendations for prescribing antibiotics apply? [0-20% / 21-40% / 41-60% / 61-80% / 81-100% of patients / DK]
- How frequently do you prescribe antibiotics to your patients for the treatment of acute pharyngitis when clinical and/or testing criteria are not met? [0-20% / 21-40% / 41-60% / 61-80% / 81-100% of visits / DK]
- How frequently do you think other clinicians in your practice prescribe antibiotics for the treatment of acute pharyngitis when clinical and/or testing criteria are not met? [0-20% / 21-40% / 41-60% / 61-80% / 81-100% of visits / DK]

Additional statements (for PC+ subjects)

[Injunctive norm] These guidelines for treating acute pharyngitis have been endorsed by the American Academy of Family Physicians, the American College of Physicians, and the Centers for Disease Control and Prevention.

Response task additions (for PC+ subjects)

- None (same as PC-)

Intervention summary (for PC+ subjects)

- During the study, you will receive regular updates on your own rate of antibiotic prescribing for patients who have non-GABHS acute pharyngitis. As a demonstration of achievable performance, these updates will also include the antibiotic prescribing rate achieved by the 10% of physicians in [name of clinic] whose prescribing rates are lowest for non-GABHS acute pharyngitis.

Additional statements (for AJ+ subjects)

[Importance of justification] Guidelines are intended to help clinicians treat the majority of their patients. However, there can be clinical reasons why a guideline might not apply to a particular patient. When there is a good clinical reason, a physician might justifiably choose not to follow a guideline.

For acute pharyngitis, antibiotics may be prescribed in guideline-consistent or guideline-inconsistent ways. In either case, a physician who decides that there is a clinically justifiable or guideline-consistent reason to prescribe antibiotics to an adult patient with acute pharyngitis should have a clear sense of what this reason is. He or she should be able to state this justification or explain how the guideline was followed.

Response task additions (for AJ+ subjects)

- To what extent do you agree with these guidelines for treating acute pharyngitis in adults? [completely agree to completely disagree]
- To what extent do you agree that antibiotics are not indicated for the treatment of acute pharyngitis in healthy patients when clinical and/or testing criteria are not met? [completely agree to completely disagree]
- (Other response tasks same as AJ-)…

Intervention summary (for AJ+ subjects)

- During the study, if you prescribe antibiotics to patient who you are seeing for acute pharyngitis, you will be asked to supply a brief written justification for prescribing antibiotics. The justification that you write will be entered in the patient's medical record. If you do not write a justification, the phrase "No justification for prescribing antibiotics was given" will appear in your encounter note.

Additional statements (for SA+ subjects)

[Reminder about alternatives to antibiotics] Regardless of whether antibiotics are prescribed for acute pharyngitis, analgesics/antipyretics can provide symptomatic relief. You can also prescribe these medications instead of antibiotics when antibiotics are not indicated. There are both over-the-counter (OTC) and prescription-only options for analgesics/antipyretics.

Even for OTC medications, writing a prescription can help your patients. In addition to serving as a reminder, writing a prescription will allow your patients to use their Flexible Savings Accounts (FSAs). Without your prescription, patients will be unable to use their FSAs to buy OTC medications.

In addition to medications, you can give patients educational materials that provide information and reassurance. You can use these materials as non-antibiotic treatments that address patients' most common concerns.

Response task additions (for SA+ subjects)

- None (same as SA-)

Intervention summary (for SA+ subjects)

- During the study, when you prescribe a medication to a patient who might have acute pharyngitis, you will be shown a list of non-antibiotic alternative prescriptions and symptomatic treatments. You will be able to select from among these treatment options, and corresponding prescriptions will be generated. You will also be able to select patient educational materials that will be printed for the patient you are seeing.
- Here is the list of non-antibiotic treatments that will be offered to you. If you want to prescribe a medication that does not appear on the list (including an antibiotic), you will be able to write this prescription as usual by closing the list. [show SA list below]

Guidelines for treating acute bronchitis in adults

- Definition

- “Acute bronchitis” refers to an acute respiratory tract infection of duration <3 weeks in which cough, with or without phlegm, is a predominant feature.

- Causes
  - Over 90% of acute bronchitis cases are <u>not</u> caused by bacteria.
  - Viruses most frequently associated with acute bronchitis include influenza B, influenza A, parainfluenza 3, respiratory syncytial virus, coronaviruses, adenoviruses, and rhinoviruses.
- Diagnosis
  - A diagnosis of acute bronchitis requires the exclusion of pneumonia. Physical examination and chest X-rays, considered in the context of specific patient and epidemiologic circumstances, are common diagnostic steps.
  - When and coughing illness last longer than 3 weeks, previously undiagnosed asthma should be considered.
  - Purulent sputum does not distinguish bronchitis from pneumonia, indicate that bacterial are present, or mean that antibiotics are necessary.
- Course of illness
  - The average duration of cough for adults with uncomplicated acute bronchitis is 2-3 weeks.
- Guideline-consistent treatments (for immunocompetent adults without complicating comorbid conditions, such as chronic lung or heart disease)
  - Treatment with cough suppressants, analgesics/antipyretics, and inhaled albuterol may relieve symptoms but not shorten the duration of illness. Elimination of environmental cough triggers (for example, dust and dander) and vaporized air treatments (particularly in low-humidity environments) are also reasonable options.
  - Provide realistic expectations for the duration of the patient’s cough, which will typically last 10 to 14 days after the office visit.
  - Routine antibiotic treatment of uncomplicated acute bronchitis is <u>not</u> recommended, regardless of duration of cough.
  - The evidence supports antibiotic treatment of patients with uncomplicated acute bronchitis <u>only</u> when there is suspicion of pertussis (i.e., when there is a high probability of exposure—for example, during documented local outbreaks). Antibiotic treatment, which does not shorten the duration of symptoms if it is initiated 7 to 10 days after onset of illness, decreases shedding of the pathogen and spread of disease. Antibiotic treatment of suspected pertussis should <u>always</u> be accompanied by a diagnostic test for public health purposes.
  - (<u>CDC recommendation link here</u>)

<u>Response tasks (for PC-/AJ-/SA- subjects)</u>

- Thinking about the patients you see in clinic for uncomplicated acute bronchitis, how often does the guideline-based recommendation against prescribing antibiotics apply? [0-20% / 21-40% / 41-60% / 61-80% / 81-100% of patients / DK]
- How frequently do you prescribe antibiotics to your patients for the treatment of uncomplicated acute bronchitis? [0-20% / 21-40% / 41-60% / 61-80% / 81-100% of visits / DK]
- How frequently do you think other clinicians in your practice prescribe antibiotics for the treatment of uncomplicated acute bronchitis? [0-20% / 21-40% / 41-60% / 61-80% / 81-100% of visits / DK]

Additional statements (for PC+ subjects)

[Injunctive norm] These guidelines for treating acute pharyngitis have been endorsed by the American Academy of Family Physicians, the American College of Physicians, the Infectious Diseases Society of America, and the Centers for Disease Control and Prevention.

Response task addition (for PC+ subjects)

- None (same as PC-)

Intervention summary (for PC+ subjects)

- During the study, you will receive regular updates on your own rate of antibiotic prescribing for patients who have uncomplicated acute bronchitis. As a demonstration of achievable performance, these updates will also include the antibiotic prescribing rate achieved by the 10% of physicians in [name of clinic] whose prescribing rates are the lowest.

Additional statements (for AJ+ subjects)

[Importance of justification] Guidelines are intended to help clinicians treat the majority of their patients. However, there can be clinical reasons why a guideline might not apply to a particular patient. When there is a good clinical reason, a physician might justifiably choose not to follow a guideline.

If a physician decides that there is a clinically justifiable reason to prescribe such antibiotics to a patient with acute bronchitis, he or she should have a clear sense of what this reason is. He or she should be able to state the justification for not following the guideline.

Response task modification (for AJ+ subjects)

- To what extent do you agree with these overall guidelines for treating uncomplicated acute bronchitis? [completely agree to completely disagree]
- To what extent do you agree that antibiotics are not indicated for the treatment of uncomplicated acute bronchitis in healthy patients? [completely agree to completely disagree]
- (Other response tasks same as AJ-)…

Intervention summary (for AJ+ subjects)

- During the study, if you prescribe antibiotics to patient who you are seeing for acute bronchitis, you will be asked to supply a brief written justification for prescribing antibiotics. The justification that you write will be entered in the patient's medical record. If you do not write a justification, the phrase "No justification for prescribing antibiotics was given" will appear in your encounter note.

Additional statements (for SA+ subjects)

[Reminder about alternatives to antibiotics]. To provide symptomatic relief to patients with acute bronchitis, you can prescribe cough suppressants, analgesics/antipyretics, and inhaled albuterol. You can prescribe these medications instead of antibiotics when antibiotics are not indicated. There are both over-the-counter (OTC) and prescription-only options for cough suppressants and analgesics/antipyretics.

Even for OTC medications, writing a prescription can help your patients. In addition to serving as a reminder, writing a prescription will allow your patients to use their Flexible Savings Accounts (FSAs). Without your prescription, patients will be unable to use their FSAs to buy OTC medications.

In addition to medications, you can give patients educational materials that provide information and reassurance. You can use these materials as non-antibiotic treatments that address patients' most common concerns.

Response task addition (for SA+ subjects)

- None (same as SA-)

Intervention summary (for SA+ subjects)

- During the study, when you prescribe a medication to a patient who might have acute bronchitis, you will be shown a list of non-antibiotic alternative prescriptions and symptomatic treatments. You will be able to select from among these treatment options, and corresponding prescriptions will be generated. You will also be able to select patient educational materials that will be printed for the patient you are seeing.
- Here is the list of non-antibiotic treatments that will be offered to you. If you want to prescribe a medication that does not appear on the list (including an antibiotic), you will be able to write this prescription as usual by closing the list. [show SA list below]

Appendix B: Post-study survey

BEARI EXIT SURVEY

1) How would you rate your overall level of satisfaction with the electronic health record (EHR) used at your clinic?
(1= Very unsatisfied, 2=Unsatisfied, 3=Neither satisfied nor satisfied 4=Satisfied 5=Very satisfied)

a) How would you rate your overall satisfaction with the alerts and clinical decision support you received for patients with acute respiratory infections?
(1= Very unsatisfied, 2=Unsatisfied, 3=Neither satisfied nor satisfied 4=Satisfied 5=Very satisfied, 0= I didn't receive alerts for acute respiratory infections.

b) How would you rate your overall level of satisfaction with the antibiotic over-prescription feedback e-mails that have been sent out?"
(1= Very unsatisfied, 2=Unsatisfied, 3=Neither satisfied nor satisfied 4=Satisfied 5=Very satisfied)

2) Based on your general experience as a clinician, please indicate how much you agree or disagree with the following statements:

i. Auditing physicians' clinical performance and providing performance feedback is an effective way to improve the quality of care (1 = Strongly agree, 2 = Agree, 3 =Neither Agree nor Disagree, 4 = Disagree, 5 = Strongly Disagree)

ii. Electronic decision support tools (e.g., reminders and alerts in your EHR) are an effective way to improve the quality of care (1 = Strongly agree, 2 = Agree, 3 =Neither Agree nor Disagree, 4 = Disagree, 5 = Strongly Disagree)

iii. Condition-specific, streamlined electronic order sets are an effective way to improve the quality of care (1 = Strongly agree, 2 = Agree, 3 = Neither Agree nor Disagree, 4 = Disagree, 5 = Strongly Disagree)

3)

d. **In the grid below, please estimate the AVERAGE time allocated to you and amount of time you feel would be needed to provide high quality care for your patients.** *(please check one box)*

Visit type	Time *allocated*	Time *needed*

v.	Complete Physical/Consultation	_______minutes	_______minutes
vi.	Routine Follow-up Visits	_______minutes	_______minutes
vii.	Urgent Care Visits (in general)	_______minutes	_______minutes
viii.	Urgent Care Visits for acute respiratory infections	_______minutes	_______minutes

***e.* Which best describes the atmosphere in your office?** *(please check one box)*	Calm, orderly		Busy, but reasonabl e		Hectic, chaotic
	□1	□2	□3	□4	□5

***f.* Please indicate how much you agree or disagree with the following statement.** *(please check one box)*	Strongly disagree	Disagre e	Neither agree nor disagree	Agree	Strongly Agree
Overall, I am satisfied with my current job	□1	□2	□3	□4	□5

**4) Please indicate how much you agree or disagree with the following statements.**	Strongly Disagree	Disagree	Neither Agree nor Disagree	Agree	Strongly Agree
a. I receive useful information about the quality of care I deliver	□1	□2	□3	□4	□5
b. When I receive a new report about the quality of care, it just makes me feel helpless	□1	□2	□3	□4	□5

c. My practice evaluates me in a way that is fair ☐1 ☐2 ☐3 ☐4 ☐5

5) *Response tasks* -- Non-specific upper respiratory infections
Click here for a brief guideline review :
http://www.annals.org/content/134/6/490.abstract

- How frequently do you prescribe antibiotics to your patients for the treatment of non-specific URIs?
[0-20% / 21-40% / 41-60% / 61-80% / 81-100% of visits / Don't Know]

- How frequently do you think other clinicians in your practice prescribe antibiotics for the treatment of non-specific URIs?
[0-20% / 21-40% / 41-60% / 61-80% / 81-100% of visits/ Don't Know]

- How frequently do you think the top 10% of clinicians in your practice prescribe antibiotics for the treatment of non-specific URIs? [0-20% / 21-40% / 41-60% / 61-80% / 81-100% of visits/ Don't Know]

- Realistically, for patients without chronic conditions, how low do you think a good doctor could get their antibiotic prescribing rate for non-specific URIs?

  [0-20% / 21-40% / 41-60% / 61-80% / 81-100% of visits/ Don't Know]
- In some online studies, a small number of participants do not pay close attention to all of the items they are answering. To indicate that you are paying close attention, please do not mark any of the choices for the following question: How frequently do you think physicians prescribe decongestants for non-specific URIs?
[0-20% / 21-40% / 41-60% / 61-80% / 81-100% of visits / Don't Know]

6) *Response tasks –Sinusitis* Click here for a brief guideline review:
http://www.cdc.gov/getsmart/campaign-materials/info-sheets/adult-approp-summary.html

- How frequently do you prescribe antibiotics to your patients for the treatment of acute sinusitis/rhinosinusitis?
[0-20% / 21-40% / 41-60% / 61-80% / 81-100% of visits / Don't Know]

- How frequently do you think other clinicians in your practice prescribe antibiotics for the treatment of acute sinusitis/rhinosinusitis?
[0-20% / 21-40% / 41-60% / 61-80% / 81-100% of visits / Don't Know]

- How frequently do you think the top 10% of clinicians in your practice prescribe antibiotics for the treatment of acute sinusitis/rhinosinusitis?
  [0-20% / 21-40% / 41-60% / 61-80% / 81-100% of visits / Don't Know]

- Realistically, for patients without chronic conditions, how low do you think a good doctor could get their antibiotic prescribing rate for sinusitis?
  [0-20% / 21-40% / 41-60% / 61-80% / 81-100% of visits / Don't Know]

7) *Response tasks*--Acute pharyngitis
Click here for a brief guideline review:
http://www.cdc.gov/getsmart/campaign-materials/info-sheets/adult-approp-summary.html

- How frequently do you prescribe antibiotics to your patients for the treatment of acute pharyngitis when clinical and/or testing criteria are not met?
  [0-20% / 21-40% / 41-60% / 61-80% / 81-100% of visits / Don't Know]

- How frequently do you think other clinicians in your practice prescribe antibiotics for the treatment of acute pharyngitis when clinical and/or testing criteria are not met?
  [0-20% / 21-40% / 41-60% / 61-80% / 81-100% of visits / Don't Know]

- How frequently do you think the top 10% of clinicians in your practice prescribe antibiotics for the treatment of acute pharyngitis when clinical and/or testing criteria are not met?
  [0-20% / 21-40% / 41-60% / 61-80% / 81-100% of visits / Don't Know]

- Realistically, for patients without chronic conditions, how low do you think a good doctor could get their antibiotic prescribing rate for acute pharyngitis?
  [0-20% / 21-40% / 41-60% / 61-80% / 81-100% of visits / Don't Know]

8) *Response tasks*-- Acute bronchitis
Click here for a brief guideline review:
http://www.cdc.gov/getsmart/campaign-materials/info-sheets/adult-approp-summary.html

- How frequently do you prescribe antibiotics to your patients for the treatment of uncomplicated acute bronchitis?
  [0-20% / 21-40% / 41-60% / 61-80% / 81-100% of visits / Don't Know]

- How frequently do you think other clinicians in your practice prescribe antibiotics for the treatment of uncomplicated acute bronchitis?
  [0-20% / 21-40% / 41-60% / 61-80% / 81-100% of visits / Don't Know]

- How frequently do you think the top 10% of clinicians in your practice prescribe antibiotics for the treatment of uncomplicated acute bronchitis?
  [0-20% / 21-40% / 41-60% / 61-80% / 81-100% of visits / Don't Know]

- Realistically, for patients without chronic conditions, how low do you think a good doctor could get their antibiotic prescribing rate for acute bronchitis?
  [0-20% / 21-40% / 41-60% / 61-80% / 81-100% of visits / Don't Know]

9) At this point in time, how would you rate your overall level of satisfaction with the e-mails that you recently received about your antibiotic prescribing?
(1= Very unsatisfied , 2, 3, 4, 5=Very satisfied)
10) How useful did you find the program in improving antibiotic prescribing practices?
(1=Not at all , 2, 3, 4, 5=Very)
11) How useful did you find the peer comparison information regarding the top performers' over-prescription rate?
(1=Not at all, 2, 3, 4, 5=Very)
12) Please read the following vignette and then answer the questions below.

Acute Bronchitis

A 27-year-old woman with no known underlying lung disease presents with a 10-day history of cough that is productive of yellow nonbloody sputum. Her symptoms began with nasal congestion and a sore throat, but these initial symptoms resolved after a few days. Her remaining symptom is the cough which is less productive than it was several days ago. She denies any known sick contacts. Her cough does not occur in long fits, and there is no post-tussive emesis. On physical examination she is not in respiratory distress, afebrile, and has normal vital signs. Lung exam is normal.

i. The probability of a major benefit from prescribing erythromycinis: [enter a number from 0-100 here] %.
ii. The probability of a minor benefit from named antibiotic (e.g., the patient feels better a day or two sooner vs. not getting the antibiotic), is: [enter a number from 0-100 here] %.
iii. The probability of a minor harm from prescribing erythromycin (e.g., temporary diarrhea or yeast infection), is : [enter a number from 0-100 here] %.

iv. The probability of a major harm from prescribing erythromycin (e.g., serious drug reaction including anaphylaxis, cardiac arrhythmia) is: [enter a number from 0-100 here] %.
v. What are the chances you would prescribe erythromycin? [enter a number from 0-100 here] %.
vi. Now imagine that the patient specifically requests an antibiotic. What are the chances you would prescribe the antibiotic? [enter a number from 0-100 here] %.

13) Are you more frequently engaging patients in antibiotics discussions since the study started?
Yes ☐
No ☐

14) Please estimate the percentage of your patients in each of these categories:

a. Suffer from chronic pain _____%

b. Have complex or numerous medical problems _____%

c. Have complex or numerous psycho-social problems _____%

d. Are generally frustrating to deal with _____%

e. Have alcohol or other substance abuse disorders _____%

15) **Please indicate how much you agree or disagree with the following statements.**	Strongly disagree	Disagre e	Neither agree nor disagree	Agree	Strongly Agree
a. Many patients demand potentially unnecessary treatments	☐1	☐2	☐3	☐4	☐5
b. Time pressures keep me from developing good patient relationships	☐1	☐2	☐3	☐4	☐5
c. I am overwhelmed by the needs of my patients	☐1	☐2	☐3	☐4	☐5

16) For a typical ARI patient, antibiotics are more likely to do harm (primarily diarrhea and yeast infections) than to do good (by speeding recovery or preventing some kind of bacterial complication).

Please rate your agreement from 1 (Low) to 10 (High). *

1 2 3 4 5 6 7 8 9 10

17) Inappropriate antibiotic prescribing to ARI patients is caused by patients' "demand" for antibiotics.

Please rate your agreement from 1 (Low) to 10 (High). *

1 2 3 4 5 6 7 8 910

18) Is inappropriate ARI prescribing caused by doctors having not enough time with patients?
Please rate your agreement from 1 (Low) to 10 (High). *
1 2 3 4 5 6 7 8 9 10

19) Do you generally support performance measurement and quality improvement for doctors' practices?

Please rate your agreement from 1 (Low) to 10 (High). *

1 2 3 4 5 6 7 8 910

20-29) Conjoint Analysis Example:

Consider each of the following Choice Pairs. For each separate Choice Pair indicate which treatment option is preferable to you. You may only indicate one choice per pair.

	CHOICE A	CHOICE B

EHR Prescribing Default Screen	ON	OFF
Peer Performance Feedback	OFF	ON
Pay for performance	$12/month	$100/month
Additional ARI Therapy Explanation Time	5 minutes per visit	1 minute per visit
MY CHOICE	X	

We will now give you 10 choice pairs and ask you to indicate your choice preference for each pair sequentially. Please pick the one of the two alternatives that you think is better.

[Choice Pairs 1 through 10]

30) Please answer the following questions:

(a) A bat and a ball cost $1.10 in total. The bat costs $1.00 more than the ball.
How much does the ball cost? _____ cents

(b) If it takes 5 machines 5 minutes to make 5 widgets, how long would it take
100 machines to make 100 widgets? _____ minutes

(c) In a lake, there is a patch of lily pads. Every day, the patch doubles in size.
If it takes 48 days for the patch to cover the entire lake, how long would it
take for the patch to cover half of the lake? _____ days

You have completed the exit survey. Thank you very much for your participation!

APPENDIX C: DETAILS OF DEVELOPMENT AND CUSTOMIZATION THAT WAS REQUIRED AT EACH SITE

Requirement/Feature	Longitudinal Medical Record	NextGen	EpicCare
**Application of eMeasure exclusion criteria from problem list, past diagnoses, and concomitant diagnoses to suppress alerts**	Not Implemented	Typical customization of existing functionality	Typical customization of existing functionality
**Justification Text Inserted into a visible portion of the medical record**	Expert programming	Expert programming	Expert programming
**Default text "No justification Provided" populating medical record**	Expert programming	Expert programming	Expert programming
**Prompt for diagnosis entry if absent at time of prescription order**	Typical customization of existing functionality	Not applicable (normal workflow)	Typical customization of existing functionality
**Custom Design Order Sets**	Typical customization of existing functionality	Typical customization of existing functionality	Typical customization of existing functionality
**Diagnosis Triggered Order Sets**	Typical customization of existing functionality	Typical customization of existing functionality	Typical customization of existing functionality
**Mechanism to remove antibiotic prescription ordered but not yet signed**	Expert programming: Medication automatically cancelled with option to reinstate with checkbox	Alerts include a custom-programmed medication management interface to remove pending prescriptions	Alert includes a link allowing user to rapidly navigate user back to the normal order interface to modify pending orders
**Accountable justifications alert stops showing**	Expert programming	After alert has been acknowledged or times out	After alert has been acknowledged or bypassed
**Alerts reappear if bypassed?**	Alerts appear for all antibiotic prescriptions at which point clinician indicates it is for an ARI.	Upon encounter sign out if antibiotic has been ordered.	Upon encounter sign out if antibiotic has been ordered.
**Development and testing**	5 months	7 months	10 months

APPENDIX D: EXAMPLE OF ACCOUNTABLE JUSTIFICATION DECISION SUPPORT

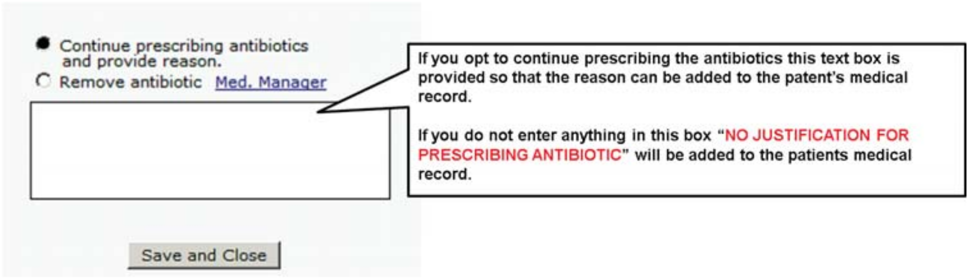

Appendix E: Diagnosis code sets used in outcome assessments and clinical decision support

This is contained in an accompanying Microsoft Excel file

Appendix F: Example of Suggested Alternatives order set

Over-the-counter medications

Decongestants

- □ Pseudoephedrine HCL (SUDAFED) 30 MG TABS
  Two tablets every 6 hours as needed for nasal congestion. Dispense 50, Refills 0.
- □ Oxymetazoline HCl (AFRIN SINUS) 0.05% SOLN
  One or two sprays in each nostril twice a day or as needed, but no more frequently than every 6 hours. Do not use for more than 3 days. Dispense 1 bottle, Refills 0.

Antihistamines

- □ Loratadine 10 MG TABS
  One tablet by mouth once a day as needed. Dispense 30. Refills 0.
- □ DiphenhydrAMINE 25 MG TABS
  Take one or two tablets by mouth every 4 to 6 hours as needed, not to exceed 6 doses in 24 hours. Dispense 24, Refills 0.

Analgesics and antipyretics

- □ Ibuprofen 200 MG TABS
  One or two tablets by mouth every 6 hours as needed for aches and pains due to colds or sore throat or to reduce fever. Dispense 50, Refills 0.
- □ Acetaminophen 500 MG TAB
  One or two tablets by mouth every 6 hours as needed for aches and pains due to colds or sore throat or to reduce fever. Do not take more than 8 pills (4000 MG) in one day. Dispense 50, Refills 0.
- □ Menthol (CEPACOL SORE THROAT) 3MG LOZG
  Allow 1 lozenge to dissolve slowly in the mouth; may be repeated every 2 hours as needed for up to 2 days. Dispense 18, Refills 0.

Cough suppressants and expectorants

- □ Guaifenesin-DM 100-10 MG/5ML SYRUP
  One or two teaspoons every 4 hours as needed for cough. Dispense 1 bottle, Refills 0.
- □ Guaifenesin 200 MG TABS
  One or two tablets every 4 hours as needed for cough. Dispense 100, Refills 0.

Prescription medications

- □ Ipratropium Bromide (ATROVENT) 0.06% SOLN
  Two sprays each nostril 4 times a day as needed for runny nose and sneezing for up to 4 days.
- □ Ibuprofen 600 MG TABS
  One tablets by mouth every 6 hours as needed for aches and pains due to colds or sore throat or to reduce fever. Dispense 28, Refills 0.
- □ GUAIFENESIN-CODEINE (CHERATUSSIN AC) 100-10 MG/5ML SYRUP
  One or two teaspoons every 4 hours as needed for cough. Dispense 180 ML, Refills 0.
- □ Benzonatate (TESSALON PERLES) 100 MG CAPS.
  One capsule every 4 hours as needed for cough. Do not take more than 6 capsules in 1 day. Dispense 30. Refills 0.
- □ ALBUTEROL HFA 108 (90 BASE) MCG/ACT AERS
  One or two inhalations every 6 hours as needed for cough. Dispense 1 inhaler. Refills 0.

Patient information (will appear in patient instructions)

☐ About Non-Specific Upper Respiratory Infection or “Common Cold”

TEXT FOR PATIENT INSTRUCTIONS

Non-Specific Upper Respiratory Infection or “Common Cold”

Your doctor has diagnosed you with a non-specific upper respiratory infection. This is also called the “common cold.” The symptoms of a cold include watery eyes, runny nose, nasal stuffiness, sneezing, scratchy or sore throat, fatigue, fever, muscle aches, and cough. Most colds last 1 to 2 weeks. Although you may feel bad, the common cold almost never causes serious illness.

Colds are caused by viruses. Cold viruses are spread through the air, through contact with people who have a cold, and on surfaces that have been touched by people with a cold. After you have caught a cold virus, it takes 2 or 3 days for you to develop symptoms. You can avoid getting and spreading colds by washing your hands frequently, avoiding other people with colds, avoiding touching your face, and coughing or sneezing into a tissue.

You cannot treat cold viruses directly, but you can treat the symptoms. Your doctor may have made specific recommendations for medications to help treat your symptoms.

In addition, you can soothe a sore throat by gargling with warm water. If you smoke, you should stop smoking and avoid smoke. You should avoid alcohol until your symptoms are gone.

Antibiotics: Antibiotics do not help colds. Antibiotics only kill bacteria, but they are not effective against viruses that cause colds. If you use unnecessary antibiotics, you run the risk of having diarrhea and yeast infections, having an allergic reaction, and increasing your risk of having an infection later with antibiotic-resistant bacteria. Colored nasal discharge or sputum is a frequent symptom of the common cold and does not necessarily indicate a bacterial infection.

You should contact your doctor if:

- Your symptoms have not improved after 14 days
- You develop a high fever (above 102°F), confusion, difficulty breathing or swallowing, severe headache, pain in your face or forehead, severe fatigue, or a rash

Appendix G: Sample Peer Comparison emails to providers

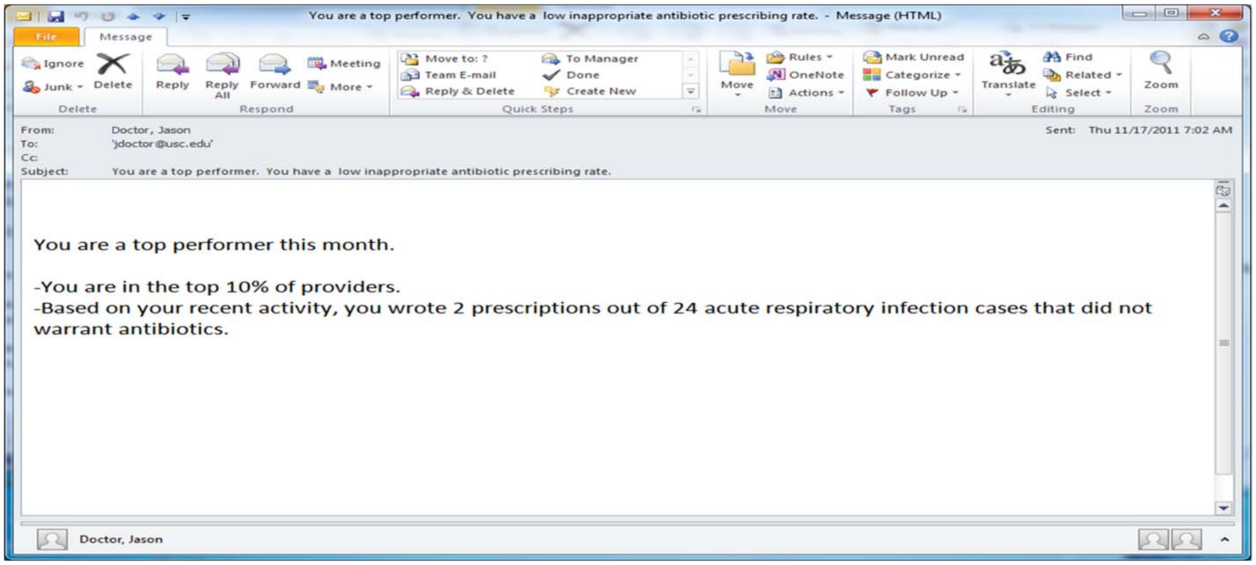

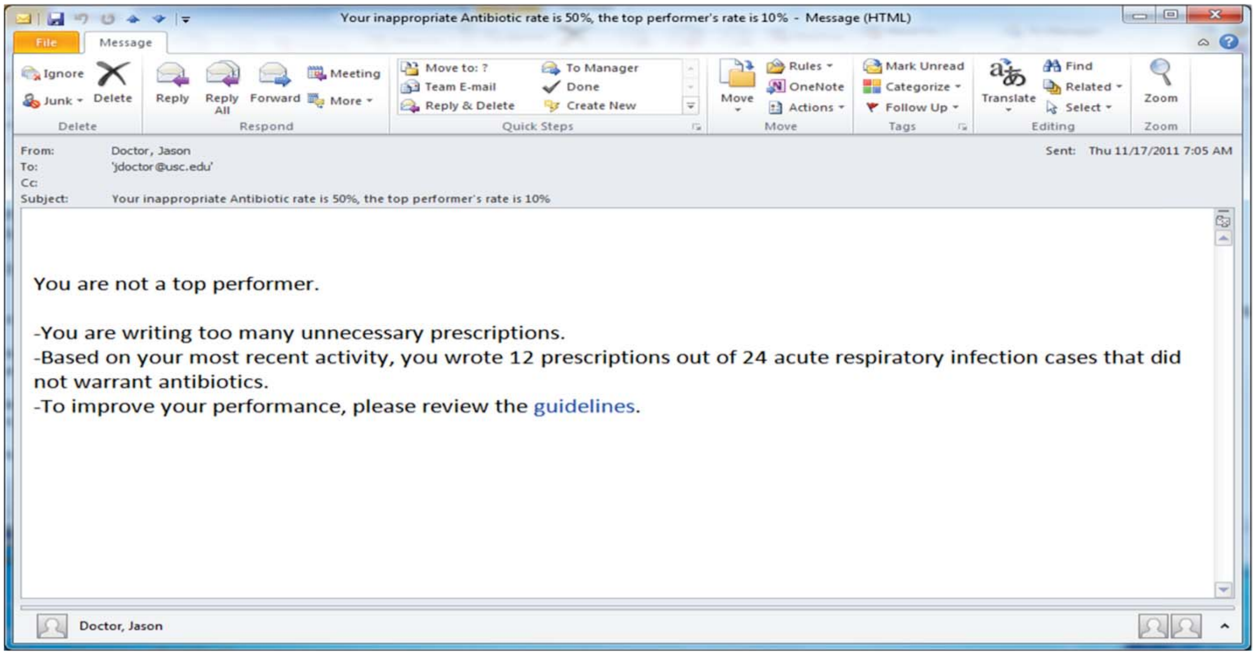

APPENDIX H: ORAL ANTIBIOTICS INCLUDED IN OUTCOME MEASUREMENTS

Cephalosporins	Other antimicrobials
Macrolides	Clindamycin
Penicillins	Linezolid
Quinolones	Telithromycin
Sulfonamides	Trimethoprim
Tetracyclines	
