# Supplementary material for: Clinician Job Satisfaction After Peer Comparison Feedback: A Secondary Analysis of a Randomized Clinical Trial
Source: JAMA Netw Open. 2023 Jun 8;6(6):e2317379. doi: 10.1001/jamanetworkopen.2023.17379 (PMC10251208; doi:10.1001/jamanetworkopen.2023.17379)
Supplement: Supplement 2. — eTable 1. Linear Model Estimating Effect of Each Intervention on Job Satisfaction eTable 2. Two-Way Interaction Linear Model Estimating Effect of Peer Comparison on Job Satisfaction When Combined With the Accountable Justification or Suggested Alternatives Intervention eTable 3. Three-Way Interaction Model Estimating Effect of Peer Comparison on Job Satisfaction When Combined With the Accountable Justification and Suggested Alternatives Intervention [file jamanetwopen-e2317379-s002.pdf]

Supplementary Online Content

Doctor JN, Goldstein NJ, Fox CR, et al. Clinician job satisfaction after peer comparison feedback: a secondary analysis of a randomized clinical trial. *JAMA Netw Open*. 2023;6(6):e2317379. doi:10.1001/jamanetworkopen.2023.17379

eTable 1. Linear Model Estimating Effect of Each Intervention on Job Satisfaction
eTable 2. Two-Way Interaction Linear Model Estimating Effect of Peer Comparison on Job Satisfaction When Combined With the Accountable Justification or Suggested Alternatives Intervention
eTable 3. Three-Way Interaction Model Estimating Effect of Peer Comparison on Job Satisfaction When Combined With the Accountable Justification and Suggested Alternatives Intervention

This supplementary material has been provided by the authors to give readers additional information about their work.

© 2023 Doctor JN et al. *JAMA Network Open.*

eTable 1. Linear Model Estimating Effect of Each Intervention on Job Satisfaction

Variable	Coefficient	Robust SE[1]	(95% CI)	t	P value
Peer comparison	0.112	0.145	(-0.181, 0.405)	0.77	0.444
Accountable justification	-0.091	0.141	(-0.375, 0.194)	-0.64	0.525
Suggested alternatives	0.034	0.144	(-0.257, 0.326)	0.24	0.813
Constant	3.711	0.116	(3.476, 3.945)	31.94	<0.001

[1] Clustered by clinic

© 2023 Doctor JN et al. *JAMA Network Open.*

eTable 2. Two-Way Interaction Linear Model Estimating Effect of Peer Comparison on Job Satisfaction When Combined With the Accountable Justification or Suggested Alternatives Intervention

Variable	Coefficient	Robust SE[1]	(95% CI)	t	P value
Peer comparison	-0.145	0.283	(-0.717, 0.427)	-0.51	0.612
Accountable justification	-0.201	0.219	(-0.642, 0.241)	-0.92	0.364
Suggested alternatives	0.063	0.157	(-0.254, 0.380)	0.40	0.690
Peer comparison x Accountable justification	0.435	0.289	(-0.148, 1.017)	1.50	0.140
Peer comparison x Suggested alternatives	0.076	0.288	(-0.505, 0.657)	0.26	0.793
Accountable justification x Suggested alternatives	-0.131	0.276	(-0.688, 0.426)	-0.47	0.637
Constant	3.785	0.123	(3.536, 4.034)	30.68	<0.001

[1] Clustered by clinic

© 2023 Doctor JN et al. *JAMA Network Open.*

eTable 3. Three-Way Interaction Model Estimating Effect of Peer Comparison on Job Satisfaction When Combined With the Accountable Justification and Suggested Alternatives Intervention

Variable	Coefficient	Robust SE[1]	(95% CI)	T	P value
Peer comparison	-0.095	0.353	(-0.807, 0.617)	-0.27	0.788
Accountable justification	-0.162	0.236	(-0.638, 0.314)	-0.69	0.496
Suggested alternatives	0.099	0.117	(-0.136, 0.335)	0.85	0.400
Peer comparison x Accountable justification	0.345	0.428	(-0.519, 1.210)	0.81	0.425
Peer comparison x Suggested alternatives	-0.006	0.477	(-0.969, 0.957)	-0.01	0.990
Accountable justification x Suggested alternatives	-0.199	0.323	(-0.851, 0.452)	-0.62	0.541
Peer comparison x Accountable justification x Suggested alternatives	0.161	0.583	(-1.016, 1.337)	0.28	0.784
Constant	3.762	0.108	(3.545, 3.979)	34.93	<0.001

[1] Clustered by clinic

© 2023 Doctor JN et al. *JAMA Network Open.*
