# Supplementary material for: Clinician Job Satisfaction After Peer Comparison Feedback: A Secondary Analysis of a Randomized Clinical Trial
Source: JAMA Netw Open. 2023 Jun 8;6(6):e2317379. doi: 10.1001/jamanetworkopen.2023.17379 (PMC10251208; doi:10.1001/jamanetworkopen.2023.17379)
Supplement: Supplement 3. — Data Sharing Statement [file jamanetwopen-e2317379-s003.pdf]

Data Sharing Statement

Doctor. Clinician Job Satisfaction After Peer Comparison Feedback. *JAMA Netw Open*.
Published June 08, 2023. doi:10.1001/jamanetworkopen.2023.17379

Data
Data available: No
